# Biological constraint, evolutionary spandrels and antagonistic pleiotropy

**DOI:** 10.1016/j.arr.2024.102527

**Published:** 2024-10-05

**Authors:** David Gems, Carina Kern

**Affiliations:** Institute of Healthy Ageing, and Research Department of Genetics, Evolution and Environment, University College London, London WC1E 6BT, UK

**Keywords:** aging, antagonistic pleiotropy, biological constraint, evolution, programmatic aging, spandrel, trade-off

## Abstract

Maximum lifespan differs greatly between species, indicating that the process of senescence is largely genetically determined. Senescence evolves in part due to antagonistic pleiotropy (AP), where selection favors gene variants that increase fitness earlier in life but promote pathology later. Identifying the biological mechanisms by which AP causes senescence is key to understanding the endogenous causes of aging and its attendant diseases. Here we argue that the frequent occurrence of AP as a property of genes reflects the presence of constraint in the biological systems that they specify. This arises particularly because the functionally interconnected nature of biological systems constrains the simultaneous optimization of coupled traits (interconnection constraints), or because individual traits cannot evolve (impossibility constraints). We present an account of aging that integrates AP and biological constraint with recent programmatic aging concepts, including costly programs, quasi-programs, hyperfunction and hypofunction. We argue that AP mechanisms of costly programs and triggered quasi-programs are consequences of constraint, in which costs resulting from hyperfunction or hypofunction cause senescent pathology. Impossibility constraint can also cause hypofunction independently of AP. We also describe how AP corresponds to Stephen Jay Gould’s constraint-based concept of evolutionary spandrels, and argue that pathologies arising from AP are *bad spandrels*. Biological constraint is a conceptual missing link between ultimate and proximate causes of senescence, including diseases of aging.

## 1 Introduction

Among animal species, lifespans vary over several orders of magnitude from adult mayflies living a mere few days to *Arctica islandica* clams living up to half a millennium (Butler et al., 2013). Thus, the process of senescence (not to be confused with *cellular senescence*, sensu Hayflick) is largely genetically determined, and a feature of the normal phenome as specified by the wild-type genome. In humans, senescence, including the manifold diseases of aging, is also influenced by diverse environmental factors that are relatively well understood. By contrast, far less is known about how the main causes of senescence arising from wild-type biological function lead to development of cardiovascular disease, cancer, chronic obstructive pulmonary disease (COPD), Alzheimer’s disease, osteoarthritis and the very numerous other evils that beset us in old age.

Our understanding of many human diseases, and the treatments based on that understanding, follow from the basic premise that disease results from disruptions of normal function, from such factors as infectious pathogens, mechanical injury, poor nutrition, toxin exposure, and gene mutation (somatic, inherited). But the main, primary cause of senescence is something quite different: it is the process of evolution. Diseases of aging are to a large extent evolutionary diseases, arising from evolutionary mechanisms that shape wild-type function (Brüne and Schiefenhövel, 2019; Nesse and Williams, 1994).

Aging and its many diseases evolve not because they provide any fitness benefit but, principally, because the force of selection against gene variants with harmful effects later in life declines with increasing age (Hamilton, 1966; Medawar, 1952). Many genes are pleiotropic, affecting multiple traits, and some show antagonistic pleiotropy (AP), i.e. in different ways both improving and impairing biological function, and fitness (Paaby and Rockman, 2013). AP genes with beneficial effects earlier in life, but pathogenic effects later can be favored by selection, thereby promoting the evolution of senescence (Williams, 1957). The role of AP in aging is now well established, and there are many examples of pleiotropic genes with benefits early in life that promote diseases of aging, including cancer, cardiovascular disease, COPD and Alzheimer’s disease (Supplementary Table 1 lists 106 potential examples of AP and related phenomena, including some related to aging) (Austad and Hoffman, 2018; Byars and Voskarides, 2020; Carter and Nguyen, 2011; Zhao and Promislow, 2019). What is largely missing, and necessary to understand the causes of aging and its many diseases, is an account of the proximate mechanisms determined by genes that exhibit AP, i.e. of the cognate evolutionary physiology (Arnold and Rose, 2023). While we understand the evolutionary (ultimate) causes of senescence, these proximate biological mechanisms of aging remain poorly defined.

In this article we explore the role of biological constraint in the evolution of aging. This topic has in the past been relatively neglected, possibly because the extent to which the characteristics of organisms are adaptive has tended to be over-estimated, relative to the determinative effects of biological constraint (Gould and Lewontin, 1979) and historicity (phylogenetic legacy) (Nesse and Williams, 1994; Williams, 1992). Our exploration is aided by the recent emergence of several principles of programmatic aging, including run-on-type and triggered quasi-programs, costly programs, hyperfunction and hypofunction (Blagosklonny, 2006; Gems et al., 2021; Kern and Stebbing, 2023; Lemaître et al., 2024; Maklakov and Chapman, 2019; Slade et al., 2024) (described below, and see Glossary). It is also supported by an expansion of the typology of biological constraint (Acerenza, 2016), to include interconnection constraint, impossibility constraint, clock constraint, mixed constraint, molecular constraint, multiplex constraint, sexual dimorphism constraint, and asynchronous and synchronous constraint. Such conceptual developments have enabled us to define several categories of ultimate-proximate mechanism involved in aging. These include AP arising from interconnection constraints (causing pathogenic costly programs and triggered quasi-programs); and AP-independent determinants arising from impossibility constraints (causing pathogenic hypofunction). This article builds upon an earlier account of AP in the context of programmatic theories of aging (Gems, 2022).

### 1.1 Defining antagonistic pleiotropy

**Figure 1:**
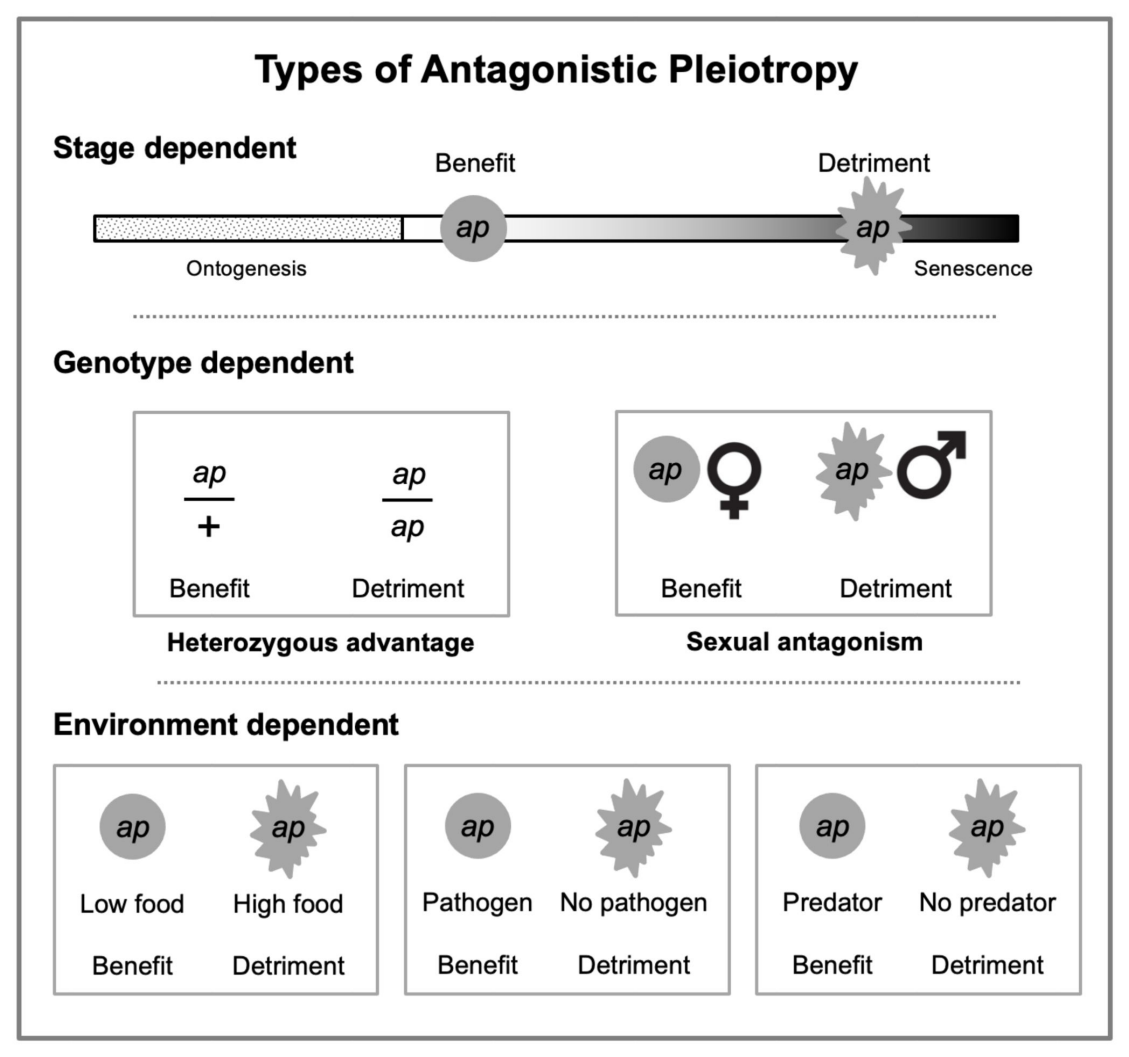
Some of the different forms of antagonistic pleiotropy. AP effects of a gene (allele), *ap*. Top, stage-dependent AP, as proposed by the evolutionary theory of aging. But detriments and benefits may occur at any point in the life history, including the same stage, and also at different locations (cell types, tissues, organs). Middle, genotype-dependent AP. Heterozygous advantage (overdominance) and sexual antagonism (intra-locus sexual conflict) are two examples, but there are others. Bottom, examples of environment-dependent AP (GxE interactions). This includes pathologies arising from mismatch between current and former environments where return to the former environment is possible.

Before considering the proximate mechanisms involved, an explicit account of AP in genetic terms is required. AP can occur when a given gene has both beneficial and detrimental effects on biological function, health and/or evolutionary fitness (Paaby and Rockman, 2013). Considered from the perspective of the evolution of aging, the term AP is often used to refer to a benefit earlier in life and a detriment in later life. But AP effects of genes can occur in many other ways, in terms of the different contexts in which benefits and detriments are manifested (Figure 1).

Benefits and detriments can occur at different points in the life history of an individual, as in *ORL1* (lectin-like low-density lipoprotein receptor 1) alleles that have been suggested to promote immune defense but also cardiovascular disease (Predazzi et al., 2013); or they can also occur at a similar time as in *AR* (androgen receptor) alleles that reduce risk of breast cancer but increase that of ovarian cancer (Levine and Boyd, 2001; Rebbeck et al., 1999). They can also occur in different individuals due to various sorts of difference in the genotypic or environmental context in which the gene is expressed. Genotypic differences include a difference in sex (i.e. intra-locus sexual conflict, or sexual antagonism) (Gavrilets and Rice, 2006), and whether the allele is heterozygous or homozygous (usually heterozygous advantage, overdominance), as in sickle cell anemia. A gene may exert beneficial or detrimental effects depending on the environment in which it is active (a form of genotype–environment [GxE] interaction), and such factors as presence/absence of pathogens, parasites and predators, food availability, and ambient temperature. For example, mutations in tumor suppressor genes can provide benefits in extreme cold (e.g. by reducing apoptosis) but this also increases cancer risk (Voskarides, 2018, 2019).

Defining AP becomes more difficult when considering a species which experiences an environmental change, such that a formerly beneficial gene becomes detrimental or vice versa. This is particularly relevant to humans, where it is thought that a number of diseases are caused or exacerbated by mismatch between modern environments and our genotype, which evolved to match the very different world of our recent ancestors. For example, increased availability of calorie rich food contributes to hypertension and type 2 diabetes (Di Rienzo and Hudson, 2005) and improved hygiene to various inflammatory diseases (Rook, 2019; Zhang and Gems, 2021). In the case of a permanent change in environment, in which an allele becomes deleterious but not beneficial, it no longer exhibits AP. However, regarding human environmental mismatch diseases, in some parts of the developing world no such mismatch exists; moreover, the wise student of recent history will regard as unsafe any assumption that mismatched features of modernity (hygiene, high food availability) are now a permanent condition for our species. Thus, for humans at least, mismatch diseases involve AP.

## 2 By what proximate mechanisms does antagonistic pleiotropy lead to senescence?

The AP theory provides an account of how senescence evolves, and predicts that wild-type genes with AP effects cause senescence. What it does not provide is detail of the biological mechanisms (say biochemical, cellular or endocrine) that actually cause aging.

**Figure 2:**
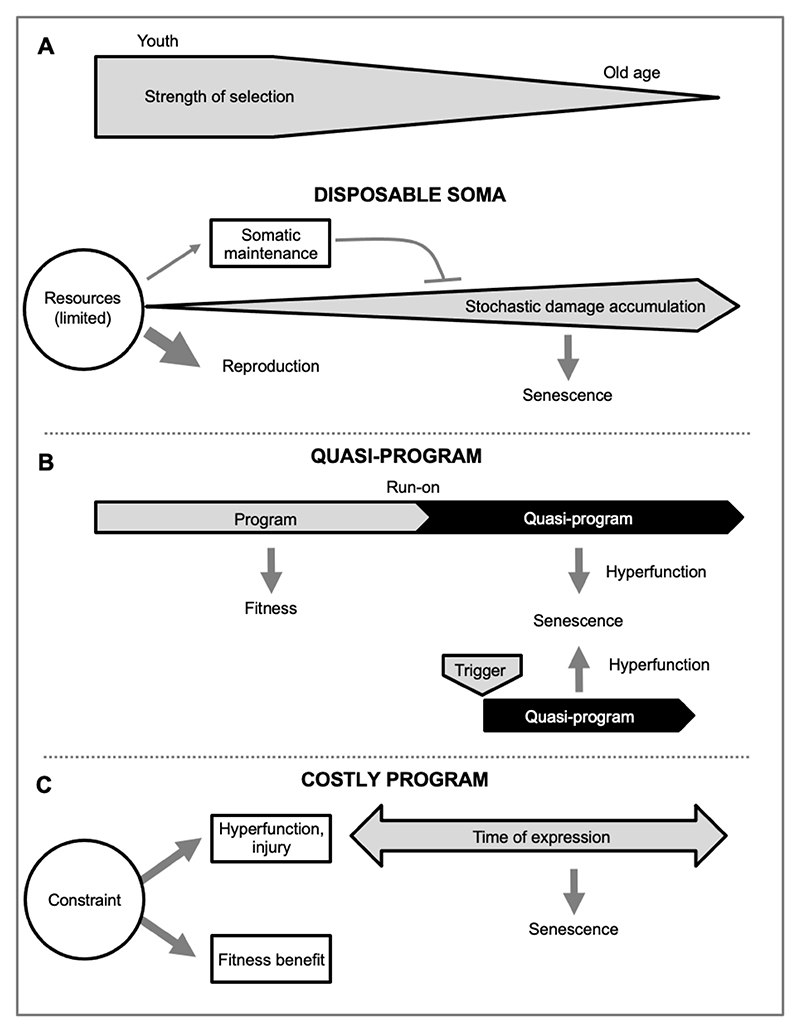
Alternative models for mechanisms by which antagonistic pleiotropy causes senescence. (A) The disposable soma theory. Here senescence is caused by stochastic molecular damage accumulation, and the inadequacy of somatic maintenance mechanisms that could prevent it (Kirkwood, 1977). Constraint results from limited resource availability to optimize both reproduction and somatic maintenance; the role of constraint in limiting somatic maintenance has been previously discussed (Suvorov, 2022). (B) Quasi-programs. Here senescence results from non-adaptive continuation or activation of wild-type biological programs (Blagosklonny, 2006). Quasi-programs may occur due to futile run-on of wild-type programs (as in presbyopia), or they may be triggered by other events (as in rheumatoid arthritis) (Kern and Stebbing, 2023). The possible role of constraint in quasi-programs is discussed below. (C) Costly programs. Here senescence results from hyperfunctional effects of programs that promote fitness, which arise due to organizational constraints. Timing of expression of costs as senescence varies; often they lie latent, and are unmasked later by wider senescence. The three models are not mutually exclusive. Costly programs are likely to be relatively more important in semelparous organisms and quasi-programs in iteroparous ones (Gems et al., 2021).

One possible mechanism is proposed by the disposable soma (DS) theory. This is based on the assumption that aging is the result of accumulated damage (particularly molecular damage) and insufficient activity of cellular maintenance processes (e.g. DNA repair, antioxidant defense). It argues that aging evolves as the result of trade-offs between resource investment into reproduction and somatic maintenance, where an optimal strategy entails levels of investment into somatic maintenance that are insufficient to prevent aging (Kirkwood, 2005; Kirkwood, 1977) (Figure 2A).

The DS theory featured strongly in biogerontological thinking in the 1990s and 2000s, and remains influential, yet it seems likely that it is of limited applicability. Empirical support for it is still limited and some evidence argues against it (Blagosklonny, 2007; Grandison et al., 2009; Piper et al., 2017; Speakman and Król, 2010; Zajitschek et al., 2019). Moreover, the theory is dependent on the damage-maintenance paradigm, and while molecular damage (particularly DNA damage) is certainly a major, primary causal mechanism in certain forms of senescent pathology (e.g. cancer), for many others, such as cardiovascular disease and type II diabetes this is far from clear (Blagosklonny, 2006; Blagosklonny, 2013; Gems and de la Guardia, 2013).

### 2.1 Programmatic mechanisms of aging: quasi-programs and costly programs

If AP is not attributable to disposable soma, then what? George Williams, who developed the AP theory, suggested one hypothetical illustration. In it, a new allele appears that promotes fitness by enhancing calcification of bone during development, but in later life causes calcification of arteries, promoting arteriosclerosis (Williams, 1957). Senescence in this example arises due to non-adaptive, futile gene action later in life.

Here senescence is programmed in the mechanistic sense (genetically determined, and involving concerted, wild-type biological activity), but not in the adaptive sense (Galimov et al., 2019). This highlights how the word “programmed” has two meanings (Mayr, 1961), whose conflation can cause confusion. To avoid this, the disambiguation *quasi-programmed* was introduced (Blagosklonny, 2006). The term *quasi-program* describes a complex, non-adaptive, development-like process arising from wild-type biological function (Figure 2B).

In Williams’ hypothetical example, a program for calcification of bone later becomes a quasi-program for vascular calcification. Recent advances in aging theory, drawing on experimental findings over the last two decades, have been exploring the possible role of programmatic mechanisms such as quasi-programs in the wild-type aging process (Blagosklonny, 2006; de Magalhães and Church, 2005; Gems, 2022; Gems and de la Guardia, 2013; Lemaître et al., 2024; Maklakov and Chapman, 2019; Slade et al., 2024).

It was long assumed that senescence arises due to a largely passive process of damage accumulation, somewhat analogous to that affecting inanimate objects such as machines. By contrast, the pathogenic effects of quasi-programs result from the *action* of biological processes, what has been described as *hyperfunction* (Blagosklonny, 2006; Gems, 2022). Hyperfunction describes a level of activity in a biological process that is higher than that which would result in optimal function and fitness, or an activity where it would be better if it were not there at all. Quasi-programs are hyperfunctional.

Quasi-programs may arise through the unregulated continuation or non-adaptive triggering of gene action later in life, and involve various forms of programmatic process (developmental, reproductive, reparative) (Blagosklonny, 2006; de Magalhães and Church, 2005; Gems, 2022). Support for the contribution of quasi-programs to senescence comes from laboratory animal-based experimental data and diverse examples of their involvement in human senescent pathology (Blagosklonny, 2006; Gems, 2024; Gems and Kern, 2022; Kern and Stebbing, 2023; Wang et al., 2018).

As a simple example, presbyopia (long-sightedness with age) results in part from the futile run-on of eye lens growth resulting in excessive lens thickening (Strenk et al., 2005). But aging-related diseases are usually more complex in etiology; more representative is rheumatoid arthritis, whose etiology involves triggered quasi-programs (Figure 2B). Here, a futile age increase in sterile inflammation (inflammaging) triggers immune cells to attack healthy joints. This transforms the surrounding tissue into a rheumatoid pannus that secretes proinflammatory cytokines, which further triggers destructive osteoclast-mediated bone resorption and loss, in a cascade of quasi-programs (Kern and Stebbing, 2023).

Another mode of programmatic aging involves costly programs, where a biological process that promotes fitness also incurs a cost. Such costs can result from various forms of hyperfunction. These include costly repurposing of somatic resources through active recycling of cellular biomass (Figure 2C). This occurs to a high degree in semelparous organisms such as monocarpic plants, but also more subtly in iteroparous ones, as in bone consumption to release calcium for lactation in mammals (Gems et al., 2021; Speakman, 2008). Costly programs also occur when defenses against an acute, potentially severe threat to health cause collateral injury to tissue, e.g. neutrophil migration which injures lung tissue, contributing to COPD (Sapey et al., 2014; Voynow and Shinbashi, 2021). This is analogous to the damage caused by the water from firefighters’ hoses when putting out a fire (*firehose-type costly program*).

A third form of costly program is enacted in social trade-offs, as in *adaptive death* (programmed organismal death, c.f. programmed cell death), a phenomenon largely restricted to organisms with a colonial lifestyle, such as colonial microbes (Galimov and Gems, 2021; Lohr et al., 2019). In adaptive death, altruistic suicide is a costly program to individuals that provides benefits at the higher, colony level; similarly, from the perspective of the dying cell, programmed cell death is a costly program that benefits the organism.

### 2.2 Hypofunction: genetically-determined deficiency that promotes senescence

The existence of a different sort of programmatic pathophysiology, distinct from hyperfunction, has been proposed (de Magalhães and Church, 2005). At the end of ontogenesis or reproduction, some aspects of biology show a slow decline, for example plasma IGF-1 levels. This, it has been argued, could reflect a form of programmatic insufficiency (de Magalhães and Church, 2005), subsequently referred to as *hypofunction* (Lemaître et al., 2024; Maklakov and Chapman, 2019; Slade et al., 2024). While it is true that many facets of living organisms show a gradual decline with age, the concept of hypofunction is problematic in certain respects, at least as initially presented.

First, in some cases decline (such as organ atrophy) is caused by active mechanisms, as where osteoporosis is caused by osteoclast hyperfunction, loss of tissue integrity due to “senescent” fibroblast hyperfunction (Campisi, 2013; Gems and Kern, 2022), or mTOR-promoted stem cell loss (Blagosklonny, 2008a). Thus, it can be difficult to distinguish between decline caused by hyperfunction from that where the initial cause is functional insufficiency. The collapse of protein folding homeostasis in young adults of the nematode *Caenorhabditis elegans* was suggested as a possible instance of hypofunction (Maklakov and Chapman, 2019). Yet this proteostatic collapse is actively promoted by germline signaling (Labbadia and Morimoto, 2015), and might serve either to unfetter protein synthetic capacity, consistent with AP, or possibly to enhance fitness by promoting adaptive death (Lohr et al., 2019). In either case, it is the product of active, programmatic function, rather than hypofunction.

Second, in contrast to hyperfunction, the causes of hypofunction evolution have remained relatively undefined, beyond being a consequence of evolutionary neglect due to the presence of the selection shadow in later life. Possible determinants of the evolution of hypofunction are discussed later in this article.

## 3 AP and biological constraint

**Figure 3:**
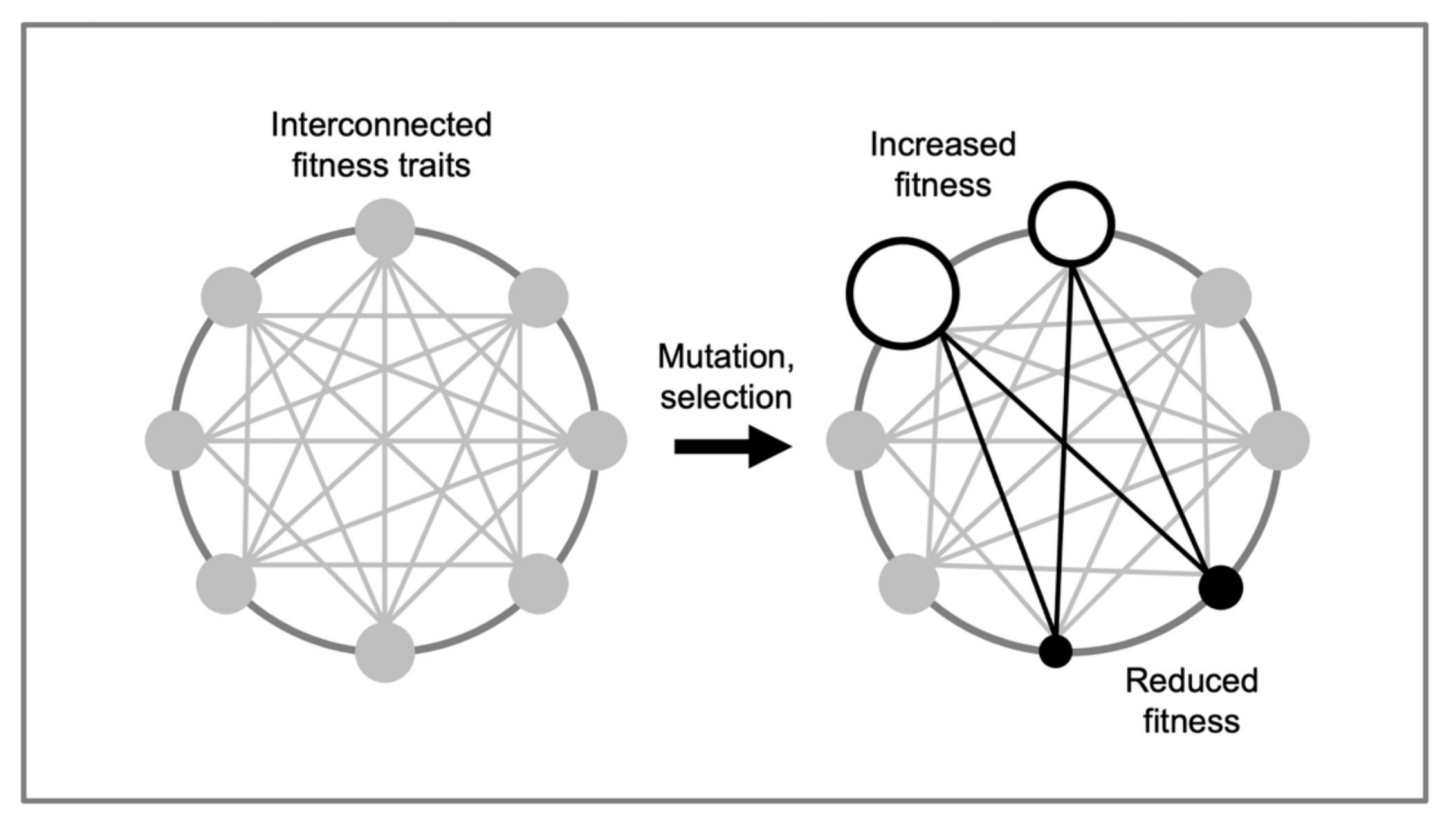
Interconnection constraints. The highly integrated nature of biological systems leads to interconnection constraint, a type of organizational constraint. Schematic representation of interconnected traits (each trait represented as a circle whose size represents fitness contribution). Here mutation followed by natural selection leads to a beneficial change in two traits (represented as increased circle size). This leads to non-adaptive, accompanying changes in two other traits that reduce fitness (reduced circle size).

The claim that AP is important in the evolution of aging implies that many genes must exhibit AP, and this is supported by the abundance of examples of AP (Supplementary Table 1). But why should so many genes have this property? The likely answer here, we argue, lies in the existence of a high degree of biological constraint, arising from the highly integrated nature of biological systems (Gould, 1997; Gould and Lewontin, 1979; Mauro and Ghalambor, 2020) (Figure 3). As Stephen Jay Gould put it, when discussing the evolution of anatomy: “any adaptive change in a complex and integrated organism must engender an automatic (and often substantial) set of architectural byproducts” (Gould, 1997). This means that a new allele that alters one trait in a way that enhances fitness can easily affect other traits adversely.

To use a simple theoretical example: for basic thermodynamic reasons increasing ATP production rate reduces ATP yield and vice versa (Pfeiffer et al., 2001). Therefore a mutation increasing ATP production rate will exhibit AP and reduce ATP yield; here ATP yield is traded off against production rate. This illustrates how AP can arise not only from properties of the molecular biology of genes or their RNA or protein products, which tend to be the focus of accounts of pleiotropy (Hodgkin, 1998; Mauro and Ghalambor, 2020; Paaby and Rockman, 2013), but also from properties of the systems that those products impact.

### 3.1 A typology of biological constraint

Key to a full understanding of AP action, we suggest, is a clear account of the nature of biological constraint. A variety of different facets of biological constraint have been described in different ways (Antonovics and van Tienderen, 1991), and enumerating these is beyond the scope of this essay. Instead, we will focus on the useful unifying scheme proposed by Luis Acerenza, that gives a systematic overview of constraint, to which the following discussion owes a great deal (Acerenza, 2016).

According to Acerenza’s scheme, two major forms of biological constraint are *selective constraints* and *organizational constraints*. Selective constraints result from ecological factors leading to different and conflicting forces of selection. As an example, males of the guppy *Poecilia reticulata* have brightly colored patches which attract females but also increase visibility to predators, such as pike cichlids (Endler, 1980). Here conflicting selection pressures act, usually on a single trait, to affect trait *presentation*.

By contrast, organizational constraints affect trait *production*. They commonly arise from the interconnected nature of biological traits, as in our prior example of ATP production rate and yield where maximizing both traits is not possible. Organizational constraints of this type, which we will refer to as *interconnection constraints* (Figure 3), are highly diverse and affect all levels of biological function, from the level of genome and biochemical pathway level, through the whole cell and organ level to the organismal and even population level. They can affect many facets of biology, including composition, kinetics, regulation and structure (Acerenza, 2016). As has been argued: “the mechanisms that cause senescence may not be mistakes but compromises carefully wrought by natural selection” (Nesse and Williams, 1994). The reason why compromise is necessary is the existence of organizational constraints involving trait interconnection.

A few illustrations follow. At the genome level the existence of genes within chromosomes means that if selection changes the frequency of an allele at a given locus, this will also affect the frequency of alleles to which it is linked (linkage disequilibrium). This can lead to genetic hitch-hiking, where selection for a beneficial allele can co-select linked alleles, including deleterious ones (Maynard Smith and Haigh, 1974). At the cellular process level, the evolution of enzyme function can be constrained in many ways, depending on whether a new reaction is catalyzed or, particularly, the same reaction with a different rate law (Acerenza, 2016). The complex topology of protein interaction networks is another potential constraint; notably, in a study of budding yeast protein interaction networks, it was observed that proteins associated with aging have greater levels of connectivity, and are more pleiotropic (Promislow, 2004; Teulière et al., 2023).

A possible example at the organelle level is membrane leak, which in mitochondria leads to energy dissipation but also reduction of production of reactive oxygen species (Brand, 2000), which can cause molecular damage. At the whole cell level there exist protein occupancy constraints, due to the fact that a given cell volume or membrane space has a limit to the amount of protein that it can contain (Acerenza, 2016). This is expected to result in competing selection pressure on different classes of proteins; this might account for observed competition in *Escherichia coli* between expression of ribosomal proteins for growth, and stress response proteins for stress resistance (Nyström, 2004). Such competition results in growth vs survival trade-offs, and AP in genes that regulate such trade-offs.

Regarding constraints acting at the organismal level, there has historically been much discussion of developmental constraints that restrict the evolution of animal morphology (Arnold, 1992; Brakefield and Roskam, 2006; Maynard Smith et al., 1985). Here a major constraint is the highly complex and interconnected process of embryonic development, including pattern formation, morphogenesis and coordinated growth. This includes constraint due to action of given signaling components (e.g. hormones and growth factors, receptors, signaling kinases/phosphatases, transcription factors) in diverse contexts (*signaling constraint*) (Mauro and Ghalambor, 2020). But of particular interest in the present context are constraints affecting not just development to adulthood, but the entire life history, encompassing ontogenesis, maturational and reproductive development during adulthood, and senescence (discussed below). At the population level, organizational constraints are expected to occur in highly social species, such as eusocial insects and colonial microbes, leading to social trade-offs. For example there is preliminary evidence that, due to the constraint of limited food availability, individual *C. elegans* constrain their reproduction in order to increase food availability for existing larvae better able to develop to the dispersal (dauer) stage before food depletion, thereby increasing colony fitness (Chapman et al., 2024; Galimov and Gems, 2020, 2021).

Acerenza’s selective vs organizational constraint categorization corresponds to Richard Lewontin’s conception of genotype and phenotype maps to connect genotype and phenotype (Lewontin, 1947). In Lewontin’s scheme, the average genotype of a population can be viewed as a point in the space of all possible genotypes (G space), and the average phenotype of the same population as a corresponding point in the space of all possible phenotypes (P space). Selective constraints reduce the *accessible* P space, by limiting the persistence of phenotypic variants. By contrast, organizational constraints restrict the P space of all possible phenotypes, because they constrain trait production.

### 3.2 Impossibility constraints

Acerenza also defines a second type of organizational constraint, using an example from enzymology. If two molecules of ethanol are chemically combined to form a single molecule of n-butanol, energy is released. This means that an enzyme that catalyzed this synthesis would be an efficient means of n-butanol synthesis, not requiring energy input. Yet no such enzyme exists. Why not? The answer may lie in the fact that the number of reaction mechanisms underlying enzyme catalysis is limited (Acerenza, 2016; Bar-Even et al., 2012). Given the existing toolbox of enzymatic mechanisms, it is simply not *feasible* for enzymatic conversion of ethanol into butanol to evolve.

**Figure 4:**
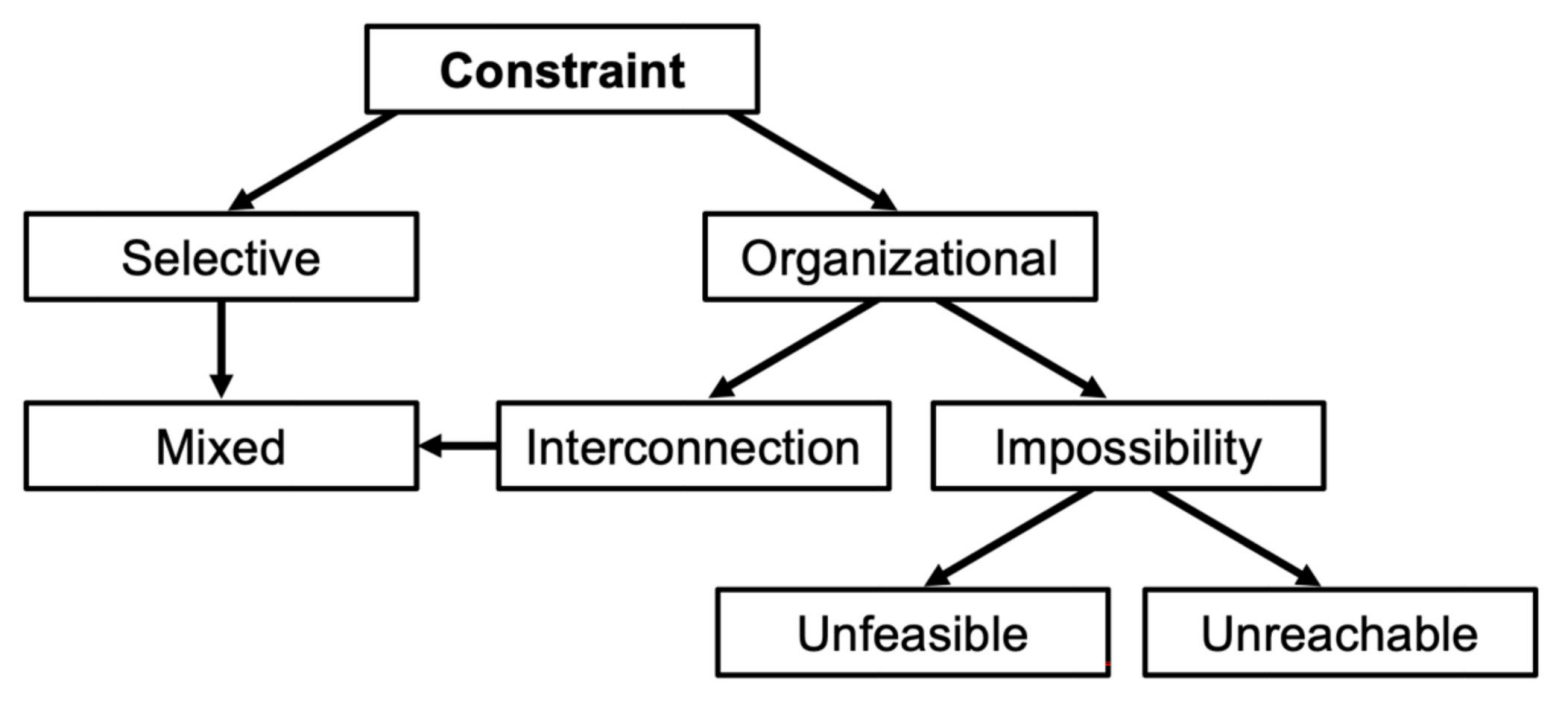
Hierarchy of major categories of biological constraint. Inspired by Acerenza (2016). Unreachability constraint includes phylogenetic constraint, in which the capacity to generate a particular phenotype has either not yet evolved or been lost (Sinervo and Svensson, 1998). Similar reasoning recently distinguished *interoperability* constraints and *type III explanations*, concepts similar to interconnection and impossibility constraints, respectively (Wensink and Cohen, 2022).

By contrast, some characters may be feasible, but evolutionarily *unreachable*, as where genes are lost from a lineage (e.g. uricase in higher primates, whose deficiency contributes to gout) (Kratzer et al., 2014). Such *impossibility constraints* are distinct from interconnection constraints, though both are organizational constraints. A major difference between them is that while interconnection constraints are expected to give rise to AP, impossibility constraints are not. However, functional defects resulting from both interconnection and impossibility constraints are programmatic in origin insofar as they are part of the wild-type phenome, specified by the wild-type genome. The hierarchy of the main forms of constraint is depicted in Figure 4.

### 3.3 Mixed constraints

Trade-offs can arise due either to selective constraints or organizational constraints. They can also arise from a combination of the two - what may be termed *mixed constraint* trade-offs. For example, considering the relationship between fertility and infection resistance, it has been suggested that immune defense not only increases resistance to infection but also the probability of spontaneous abortion (Westendorp, 2015). Given that half the genes in an embryo come from the father, they are foreign to the mother’s immune system and, as a result, the embryo is vulnerable to immunological rejection. The constraint operative here is that on optimizing both fertility, an internal function, and resistance to infection, an external, environmental factor (like the above-mentioned guppy-eating pike cichlids).

## 4 How constraint causes aging

This brings us to the main question in this article. Accepting that biological constraint is a cause of AP in general, what about AP that contributes to senescence? Does biological constraint play a role here, and if so at what level(s) of organization?

The type of trade-off by which AP is thought to promote aging is the life history trade-off, where mutations have a positive effect on fitness at one, earlier stage in the life history, but a negative effect at another, later stage (Acerenza, 2016; Sinervo and Svensson, 1998; Zera and Harshman, 2001). Yet what remains unclear is how constraint might lead to life history trade-offs, including senescent changes. The preceding discussion also raises a number of new questions. Biological constraint can generate AP, but where constraint leads to aging does this inevitably involve AP? And is AP always a consequence of constraint? Are quasi-programs a consequence of constraint? (We argue below that the answers here are, respectively, no, no and sometimes). The following sections make a first attempt at answering these new questions.

### 4.1 How do constraints promote aging?

How might constraint give rise to AP in *programmatic* aging? A fundamental form of constraint giving rise to life history trait trade-offs relates to allocation of resources. Here different fitness components may compete for limited resources (e.g. food), or simultaneous maximization may be impossible, as for example with egg size and brood size in birds (Guillaume and Otto, 2012; King et al., 2010; van Noordwijk and de Jong, 1986). The disposable soma theory provides a possible account of how resource availability constraint could contribute to AP in *damage-maintenance* mechanisms of aging, as previously discussed (Kirkwood and Rose, 1991; Lemaître et al., 2015).

One possibility is that such constraints involve differential resource allocation but of a different sort to the disposable soma theory. Costly programs are active at high levels in semelparous organisms that die as the result of suicidal reproductive effort, such as Pacific salmon and rice plants (Gems et al., 2021). Here, rapid senescence is due, at least in part, to breakdown of tissues and organs to provide resources for reproduction. This mechanism does involve a disposable soma of a kind, but not in the sense meant in Kirkwood’s theory. The harm here is from active self destruction, rather than failure to invest in protection against molecular damage. Such costly programs exemplify aging where AP results from interconnection constraint. It is not possible to maximize simultaneously both autophagic processes to support reproductive effort, and retention of somatic biomass (Gems et al., 2021).

The presence of a different type of programmatic constraint can be deduced from the existence of programmatic rate-of-living or methylation clock effects (de Magalhães, 2012; Gems, 2022; Gems et al., 2024; Raj and Horvath, 2020). At higher temperatures, fruit flies develop faster and reproduce sooner (benefit) but also senesce faster (cost). Thus, faster development is traded off against faster aging. Here the existence of the program (possibly running on into quasi-program) is itself a constraint; but, critically, the operative constraint is that increasing program play rate promotes both fitness and aging. In other words, the relationship between clock speed and temperature is fixed: there is a *clock speed constraint* (Gems et al., 2024).

### 4.2 Developmental constraint and quasi-programs

Returning to Williams’ AP scenario of a Ca^2+^ deposition program that promotes bone growth but then vascular calcium deposition (Williams, 1957): does constraint play a role in this case? Here it would appear that, owing to the late-life selection shadow, the program of calcifying bone is simply left running with pathogenic consequences. Thus, one might conclude that constraint plays no role. By the same token, quasi-programs resulting from complex program run-on (Blagosklonny, 2006) would seem not to involve biological constraint either. Yet on closer inspection, the Ca^2+^ deposition gene can be seen to be subject to interconnection constraint of a sort not discussed hitherto.

The aging process includes mechanisms of a developmental nature, as in ontogenesis but, more plausibly, developmental processes of adulthood, as in reproductive and regenerative processes (de Magalhães and Church, 2005; Gems, 2022; Gems et al., 2024; Maklakov and Chapman, 2019). Developmental processes are subject to constraint. Consider the famous example of the panda’s thumb, where selection for evolution of an additional digit in the hand (forepaw) led to non-functional evolutionary changes in the equivalent bones of the foot (hind paw) (Gould, 1991). In this case, an interconnection constraint is operative, due to similar developmental processes determining morphology in forepaws and hind paws. Here the constraint, like that linking ATP production rate and ATP yield, acts at the same time (synchronously) on hand and foot development.

But the process of development involves traits that are separated in time as well as in space. This suggests that interconnection constraints are likely to exist between processes operative at different stages in ontogenesis, and in later, adult developmental (or *maturo-developmental*) processes (Gems et al., 2024). In other words, it is likely that there exist *asynchronous* as well as synchronous developmental constraints.

**Figure 5:**
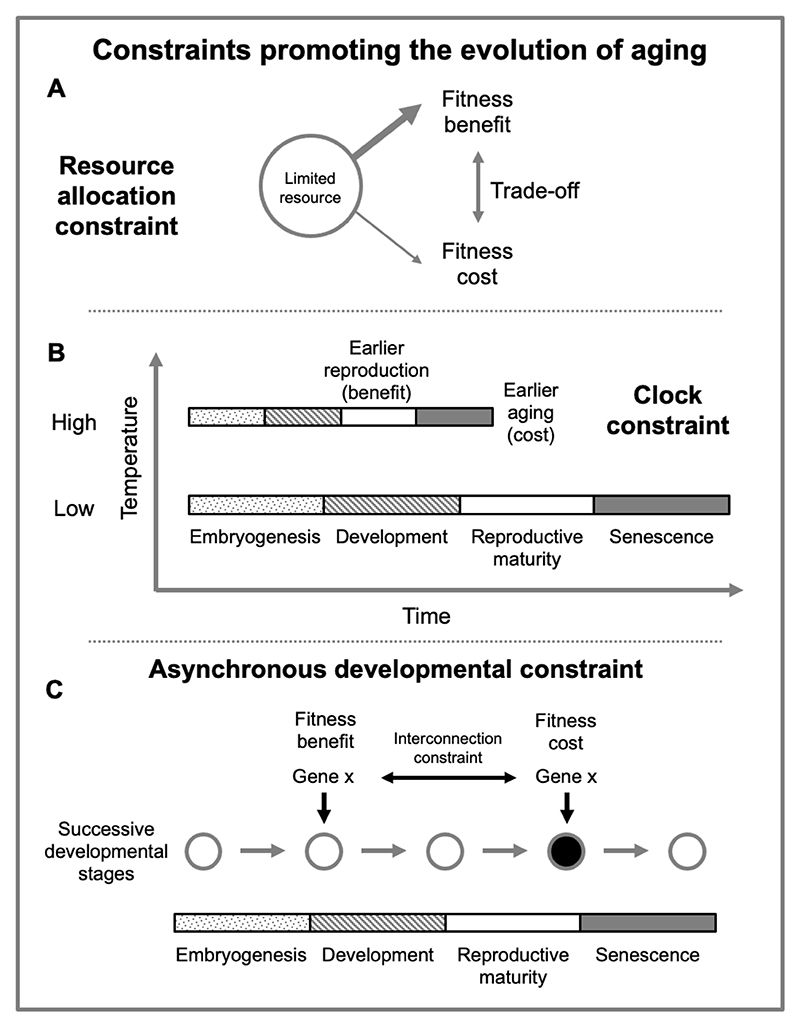
How constraint can cause programmatic senescence. (A) Resource allocation constraint, e.g. costly programs, disposable soma. (B) Clock speed constraint, where rate of the entire development and aging process is altered e.g. by changes in ambient temperature (poikilotherms only), and perhaps altered growth regulators (e.g. GH, mTOR), and evolutionary change. The constraint is imposed by the necessary relationship between biological process rate determinants (e.g. temperature) and biological process rate. (C) Asynchronous developmental constraint, where developmental effects of a process (e.g. gene action, such as Ca^2+^ deposition) provide a fitness benefit at an early developmental stage, but a fitness cost at a later stage. Dark fill denotes deleterious change.

This would be consistent with observed pleiotropic effects of genes at different stages in development. For example, in *C. elegans* the DAF-4 TGF-β/BMP receptor inhibits entry into dauer diapause early in larval development, and then directs male tail development in later larval stages (Estevez et al., 1993). In mammals, genes orchestrating development and morphogenesis act at multiple times in development and may therefore be subject to asynchronous developmental constraint. As an example, the morphogen sonic hedgehog (SHH) contributes to the development of deciduous (milk) and permanent teeth at different stages in development (Hosoya et al., 2020). Different possible ways in which interconnection constraint could contribute to the evolution of aging are summarized in Figure 5.

The existence of asynchronous developmental constraint, leading to trade-offs and AP, provides an interesting perspective for considering the possible evolutionary origins of quasi-programs. Here a sequence of developmental stages creates a series of distinct biological contexts in which a given biological function (e.g. gene action) impacts fitness. Returning to Williams’ Ca^2+^ deposition gene, this suggests a scenario in which a context emerges in late life in which the increased activity of this gene is pathogenic. Here the Ca^2+^ deposition quasi-program is *triggered*, rather than being a consequence of run-on. This argues that asynchronous developmental constraint promotes evolution of triggered quasi-programs but not run-on-type quasi-programs.

According to this scenario (Figure 5C), during aging a context appears in which the Ca^2+^ deposition gene becomes pathogenic. Interconnection constraint prevents optimization of the Ca^2+^ deposition gene activity level in both contexts. A virtue of this account is that, as outlined above, triggered quasi-programs seem to play a greater role than run-on-type quasi-programs in the etiology of late-life diseases (Gems, 2024; Kern and Stebbing, 2023).

**Figure 6:**
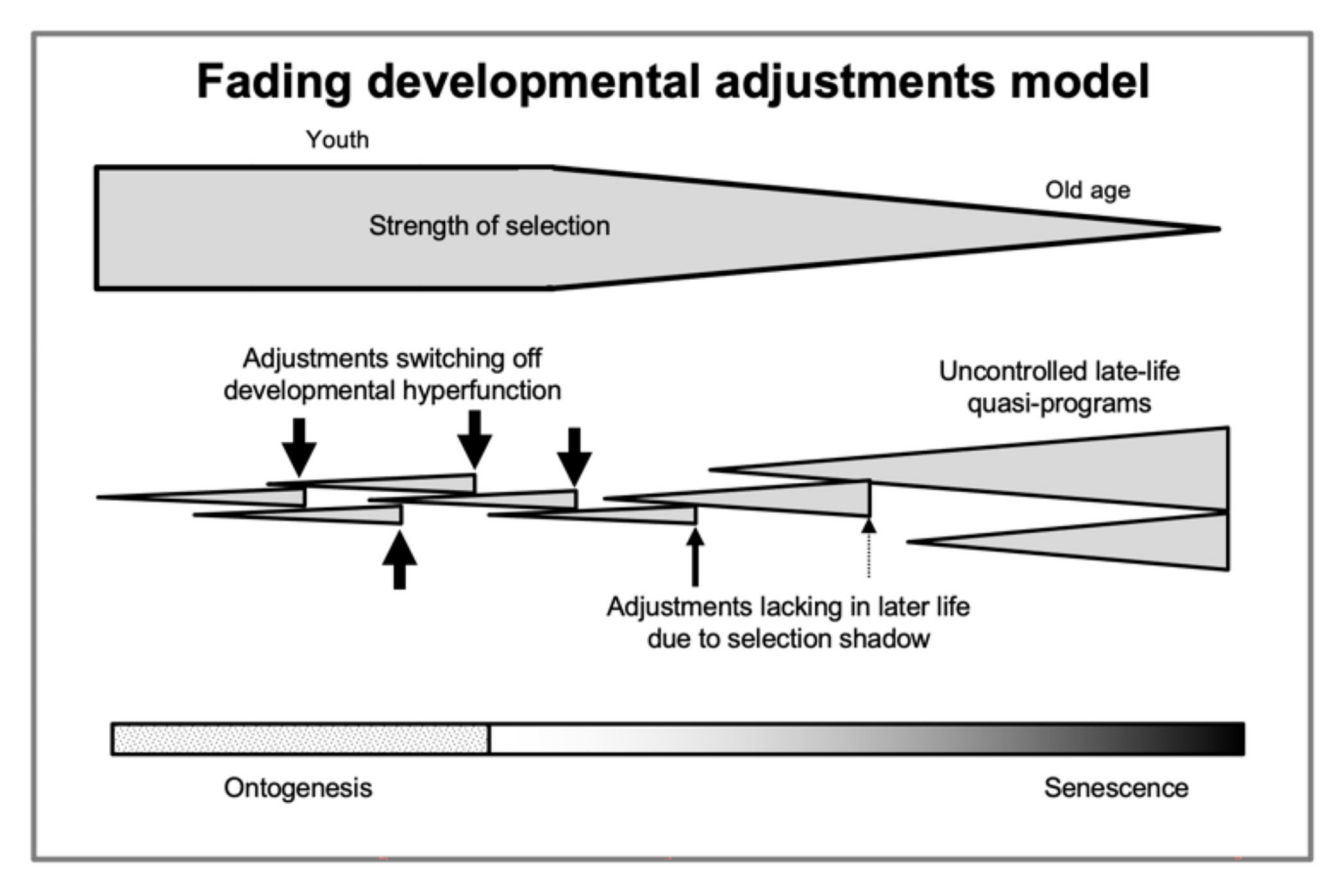
Fading developmental adjustments model. Hypothetical scheme. During ontogenesis, diverse mechanisms of programmatic adjustment prevent run-on of program into quasi-program, and shape normal development. In later life, due to the selection shadow, such adjustment mechanisms fade away. Cf the absent late-life off switch for Ca^2+^ deposition in Williams’ hypothetical example (Williams, 1957). Developmental programs are depicted as triangles, with increased maximum triangle height (right) denoting quasi-programs.

Viewing Williams’ Ca^2+^ deposition gene scenario in the light of the programmatic theory provides one further, rather striking perspective. He argued that a mechanism to suppress the pathogenic effect by switching the Ca^2+^ gene off in later life could in principle evolve, but this may not occur due to lack of late-life selection (Williams, 1957). Viewing development and aging as related phenomena: ontogenesis is a process of many corrections, where developmental trajectories are shaped by an orchestrated series of developmental-genetic adjustments to generate the final, adult phenome. Williams’ missing off-switch idea suggests a scenario where diverse quasi-programs emerge due a deficiency of later developmental adjustments (Figure 6). This deficiency is a consequence of the selection shadow and asynchronous developmental constraint.

A final point about asynchronous vs synchronous constraint: while the former will generate trade-offs and AP that are asynchronous, the latter can generate asynchronous as well as synchronous ones. This is because costs may remain latent and only emerge to affect fitness later in life. For example, rapid pumping of the pharynx in young *C. elegans* adults optimizes food ingestion, but may also cause mechanical damage to the pharyngeal cuticle. This increases susceptibility to infection that spreads later in life, probably due to wider organismal senescence, and increases late-life mortality (Zhao et al., 2017).

### 4.3 Evolutionary origins of hypofunction

The term *hypofunction* was coined by Alexei Maklakov and Tracey Chapman to describe an inherent, programmatic insufficiency of function that contributes to senescence (de Magalhães and Church, 2005; Maklakov and Chapman, 2019). From the above account of biological constraint, it is possible to deduce two different ways in which hypofunction could evolve, the first involving interconnection constraint, and the second impossibility constraint.

Regarding interconnection constraint: if a given function produces a cost and a benefit, and selection to reduce the cost is greater than that to promote the benefit, a deficiency in the latter may result, i.e. hypofunction. This can be illustrated by AP exhibited by the *AAT1* gene. This encodes α1-antitrypsin which inhibits neutrophil elastase. Migration of neutrophils to sites of infection in the lung causes tissue damage, including breakdown of elastin, which during aging can contribute to COPD (Sapey et al., 2014; Voynow and Shinbashi, 2021).

The rare *AAT1* Z allele reduces AAT levels, which elevates elastase activity, potentially increasing COPD risk, but also protects against myocardial infarction (Listì et al., 2007). It has been suggested that the latter occurs because neutrophil elastase breaks down elastic tissue in the arterial wall, altering the distensibility of the vessel wall in a way that reduces blood pressure and cardiac load (Dahl et al., 2003). Thus, the harm to cardiovascular health arising from wild-type *AAT1* is, according to the Dahl et al. hypothesis, deficiency in a corrective mechanism that would otherwise reduce hypertension, i.e. hypofunction.

That interconnection constraints can give rise to both hyperfunction and hypofunction is amply illustrated by a survey of actual and potential examples of AP (Supplementary Table 1; Supplementary Discussion). For particular genes that exhibit AP, it is not uncommon for multiple different forms of AP to be present, involving distinct forms of constraint and programmatic mechanisms. That a given gene can be subject to such *multiplex constraint* is consistent with the highly integrated nature of biological systems, particularly in metazoa. Multiplex constraint can be understood as a cause of high-dimensional pleiotropy (Wensink and Cohen, 2022).

Regarding impossibility constraint: here, functional deficiency in later life occurs not as a cost linked to an earlier benefit, but because of inability of the function to evolve. As possible examples of hypofunction of this type, we offer the aging of elephants’ teeth, and the human menopause.

The jaws of elephants have, on average, one massive molar on each side. As these molars are worn down, they are replaced by new ones developing from the back of the jaw and moving horizontally forward (successional teeth). Elephants can live up to about 75 years, during which time each set of molars is replaced five times (Lee et al., 2012). Feeding in elephants surviving into their eighth decade is increasingly impaired as their last teeth wear down, leading to starvation and death (Finch, 1990).

But why in old elephants does odontogenesis stop? One possibility is weakened selection in late life; yet this seems unlikely given that some elephants in the wild die as the consequence of their inability to produce new teeth. During the evolutionary emergence of elephants there occurred a switch from all adult teeth emerging at once to the molars appearing successionally. Thus, elephant longevity is promoted by their eking out one at a time their limited stock of premolars and molars. The occurrence of death from starvation due to tooth loss in elderly elephants suggests that the nature of the odontogenetic developmental program is an unsurmountable constraint to the evolution of further rounds of tooth replacement, i.e. an impossibility constraint.

Elderly elephants become toothless due to an odontogenetic program with a clear endpoint due to depletion of a limit resource (here tooth buds). This is analogous to a sand clock (hourglass), where the number of grains of sand is a determinant of the time at which the clock reaches its end.

A similar sand clock-type depletion mechanism appears to be operative in the human ovary, and contributes to the timing and onset of menopause. One cause of menopause is depletion of oocyte stocks that form prior to birth; by contrast spermatogenesis continues throughout adulthood in males. This depletion is accelerated by a rapid age increases in the rate of oocyte apoptosis (follicular atresia), such that increasing initial oocyte stock size would be expected to delay menopause only marginally.

Notably, females of other higher primates keep reproducing until late in life, and reproductive span in women is little different to those of chimpanzees; the age at last birth in female chimpanzees and women is 42 and 45, years respectively, though maximum lifespans are 53.4 and ~110 years, respectively (Robson and Wood, 2008). It has been argued that the human menopause evolved not because it promotes fitness, but because as hominin longevity increased, production of a longer reproductive span in hominin females was simply not possible (Austad, 1994; Marlowe, 2000). According to this view, menopause results from an impossibility constraint.

**Figure 7:**
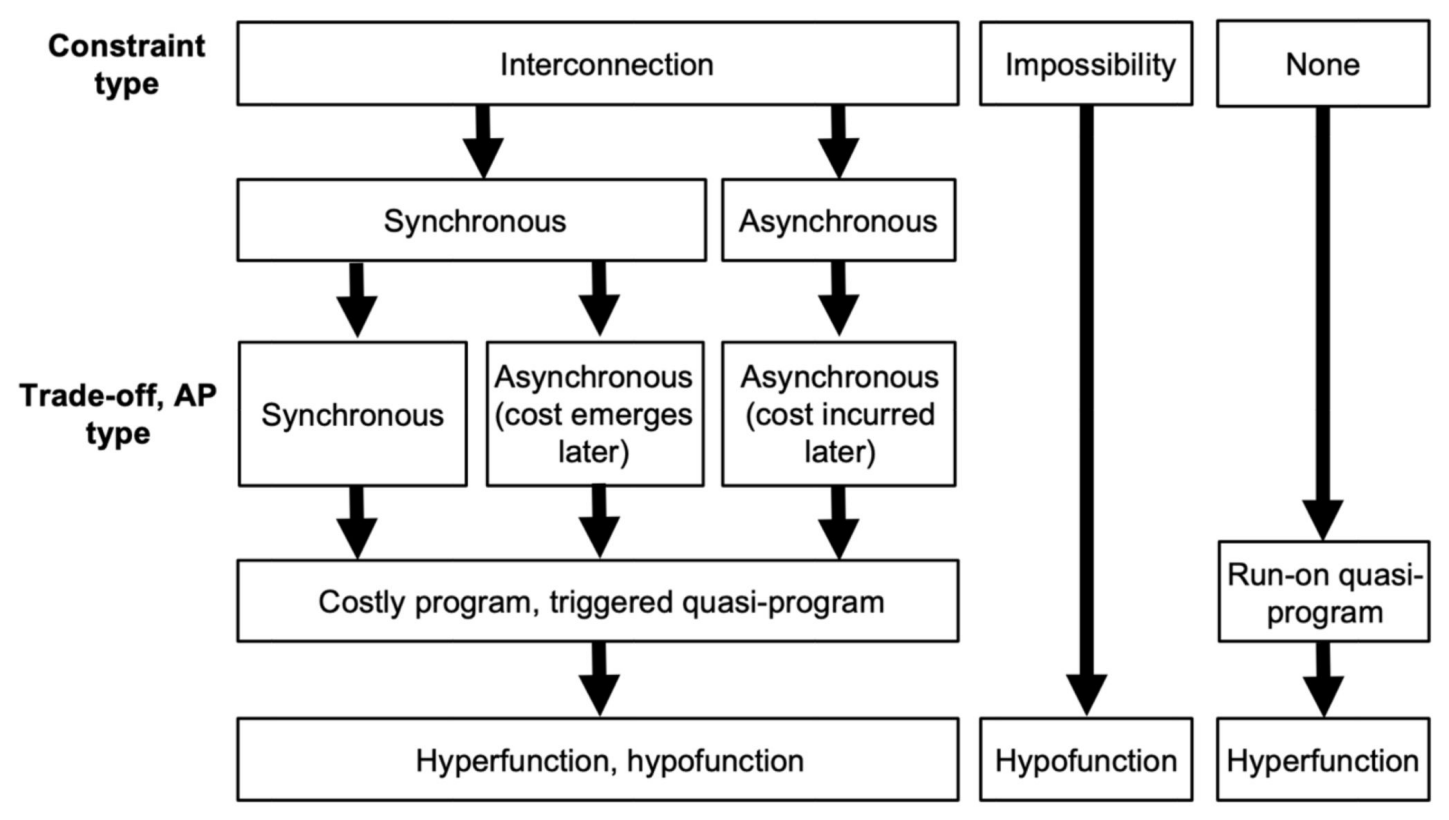
Biological constraint and programmatic mechanisms of aging. Simplified overview. The possibility that run-on-type quasi-programs can be a consequence of constraint may warrant further consideration.

In these examples, it can be seen that hypofunction resulting from impossibility constraint is programmatic insofar as it is, in a broad sense, an outcome of the wild-type genome. Yet, in contrast to hyperfunction, it is not that wild-type gene function causes it to happen. One can say that the presence of fins on a goldfish is a consequence of gene action, but one cannot say the same of their lack of wings. By the same token, phenotypic insufficiency caused by hypofunction is not the result of wild-type gene hyperfunction. The absence is, in a manner of speaking, a non-adaptive design feature. An overview of how biological constraint can lead to distinct modes of programmatic aging is shown in Figure 7.

## 5 Constraint, AP and aging in a wider context

### 5.1 AP and the spandrels of San Marco

**Figure 8:**
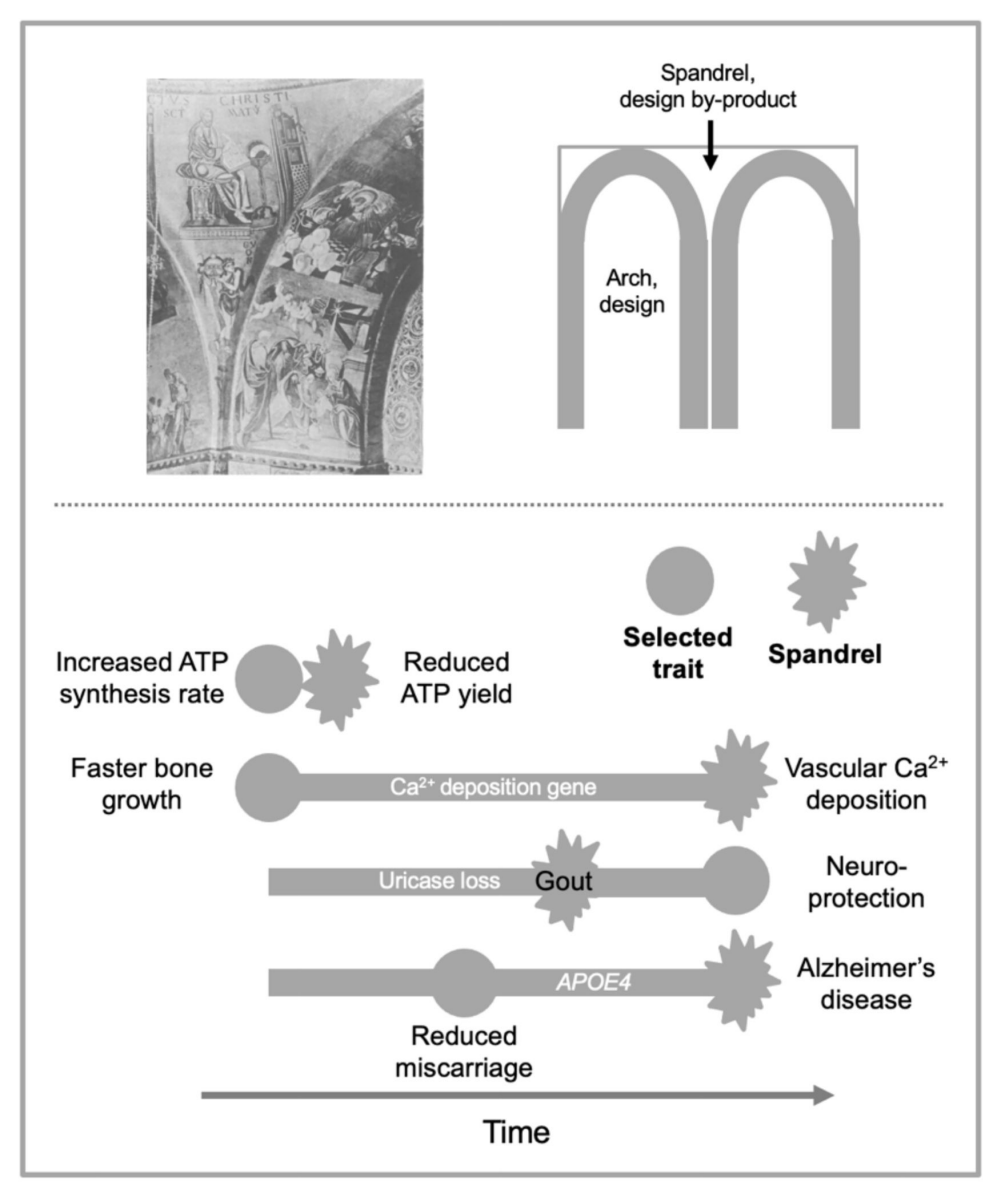
Constraint, AP and the spandrels of San Marco. Top, the spandrels concept. Left, decorated spandrels, similar to those under the dome of St. Mark’s Basilica which Stephen Jay Gould reflected upon during a visit to Venice (Gould and Lewontin, 1979). The spandrels shown here are from Monreale Cathedral, Sicily, and bear depictions of the story of Noah’s ark; those in St. Marks are decorated with depictions of holy figures. Right, a spandrel is the area between the tops of two adjoining arches. Here, the architect’s purpose is to create two arches; the spandrel appears as an unintended consequence (cf. pleiotropy). Bottom, four possible examples of spandrels arising from biological constraint, involving AP, and varying in terms of relative timing of benefit and cost. First: ATP synthesis (hypothetical example for illustration purposes). Here increasing ATP synthesis rate synchronously reduces ATP yield (spandrel) due to interconnection constraint. Second, Williams’ Ca^2+^ deposition gene example (Williams, 1957). Here a developmental change enhancing bone growth early in life promotes arteriosclerosis later in life (spandrel), due in principle to an asynchronous developmental constraint. Third, uricase gene loss. Here, absence of uricase is neuroprotective (Hong et al., 2015; Lu et al., 2016; Pakpoor et al., 2015; Weisskopf et al., 2007) but also hypofunctional in that it leads to gout (spandrel) due to urate accumulation. Fourth, the *APOE4* allele reduces miscarriage (van Exel et al., 2017) but increases late-life disease, including Alzheimer’s disease (spandrel). Note that run-on quasi-programs may not be the product of constraint, yet as “incidental consequences of evolutionary change” they are spandrels.

A cogent discussion of the importance of biological constraint in evolution is Gould and Lewontin’s influential essay “The spandrels of San Marco and the Panglossian paradigm: a critique of the adaptationist program” (Gould and Lewontin, 1979). This uses an architectural analogy to explain how organizational constraint can lead to the generation of new traits. In architecture, a spandrel is the approximately triangular space between the shoulders of adjoining arches and the ceiling above (Figure 8). Spandrels arise as a secondary consequence of the construction of adjoining arches due to geometric constraint, rather than from any intention of architects to create spandrels. Similarly, due to the presence of organizational constraints, selection for new traits can generate spandrel-like secondary changes.

Likely examples of evolutionary spandrels mentioned by Gould include the presence in mammals of nipples on males and the clitoris in females, the rudimentary thumb on the foot of pandas, the umbilicus of snails, and various miracles of human cognition (Gould, 1980, 1991, 1997). Male nipples and, seemingly, the clitoris are a consequence of what one may call *sexual dimorphism constraint*. This arises from the fact that males and females of a given species share a common embryogenetic process. Consequently trait changes in one sex can easily bleed through to the other (male nipples from the program for mammary gland development, and the clitoris from that of penis development). This constraint is important in the evolution of human aging; for example, human longevity likely evolved due to selection for increased late-life reproduction in men and, due to the sexual dimorphism constraint, is also present in women (Gems, 2014; Marlowe, 2000). According to this view, post-menopausal longevity in women is a benign evolutionary spandrel.

Let us return once more to ATP synthesis, and the relationship between organizational constraint and AP. As discussed, increasing ATP production rate reduces ATP yield and vice versa; thus, a hypothetical new allele, favored by natural selection because it increases ATP production rate would also reduce ATP yield, thus exhibiting AP. Notably, the reduction in ATP yield is an evolutionary spandrel. In fact, in all examples of AP resulting from constraint, the cost coupled to the benefit arises from a spandrel. The depiction of interconnection constraints in Figure 3 for instance also includes spandrels. Antagonistic pleiotropy is the spandrel principle viewed through the lens of evolutionary genetics.

Regarding AP as a cause of aging, a notable difference to Gould’s architectural analogy is that the selected and unselected traits are separated in time in the former and space in the latter. We postulate that the main form of constraint operative in the delayed onset spandrels through which AP causes aging is asynchronous developmental constraint.

According to this discussion, forms of senescence that arise from constraint (Figure 7), and the diseases that they cause, are noxious *bad spandrels*. Williams approved of the architectural analogy, defining a spandrel as “a structure arising as an incidental consequence of some evolutionary change” (Williams, 1992), yet seemingly did not register its relevance to AP. In his Ca^2+^ deposition gene example, the accumulation of Ca^2+^ in blood vessel walls is a bad spandrel (Figure 8). Late-life diseases arising from programmatic aging can be understood as bad spandrels (particularly insofar as they arise from interconnection constraint). Osteoarthritis, COPD, prostate cancer, Parkinson’s disease: plausibly these are all to an extent the spandrels of San Marco, but decorated with demons and skeletons rather than angels and saints.

### 5.2 Constraint and programmatic damage

Recent discussions emphasizing the importance of programmatic mechanisms view the accumulation of molecular damage as a relatively minor determinant of the aging process, at least in terms of its primary causes (Blagosklonny, 2008b; de Magalhães and Church, 2005; Gems, 2022; Maklakov and Chapman, 2019). Accepting that molecular damage accumulation does contribute to aging to some extent, one may ask: what is the relationship between constraint, AP and programmatic mechanisms on the one hand and molecular damage accumulation on the other?

**Figure 9:**
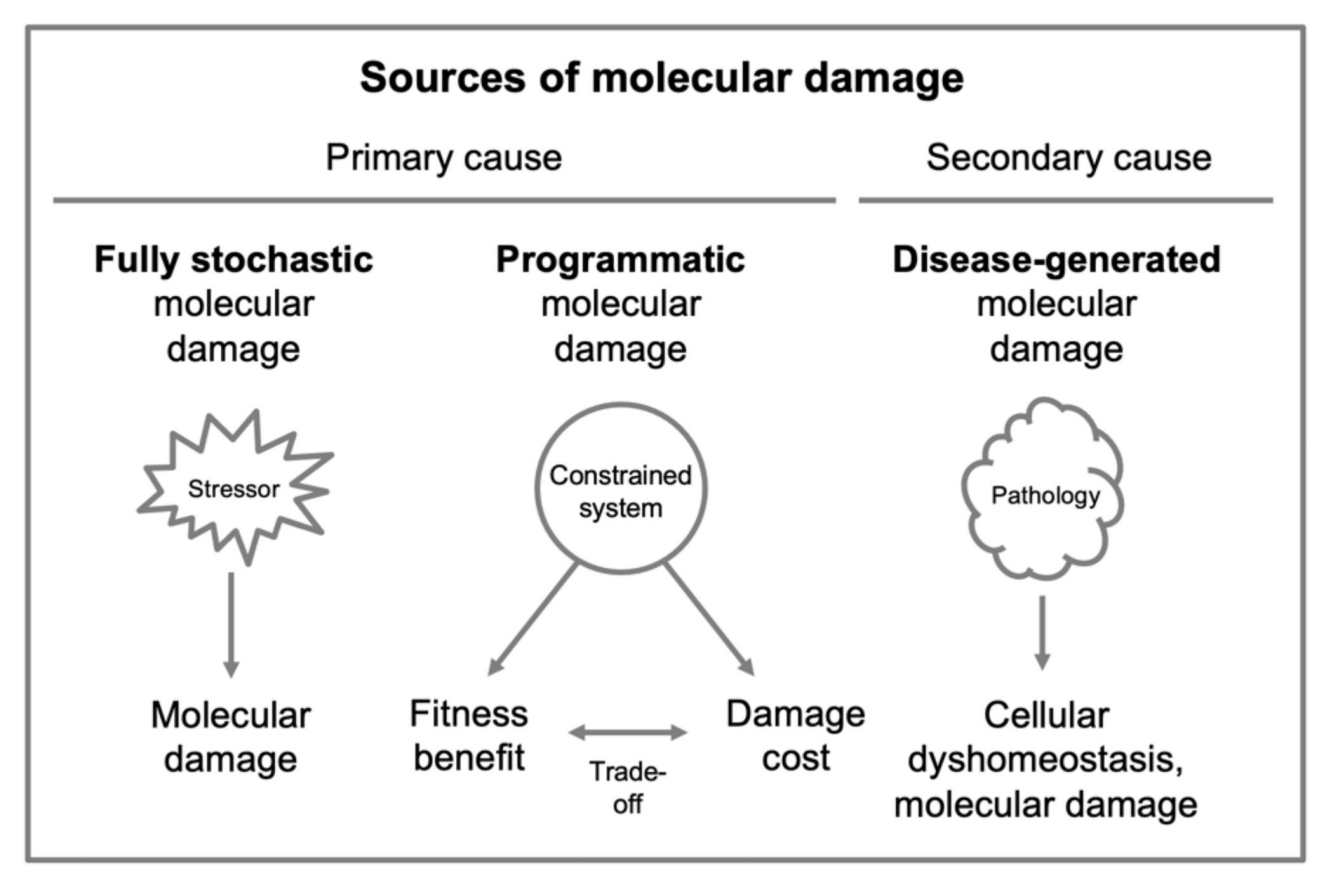
Programmatic molecular damage. Three sources of molecular damage accumulation that contribute to senescence. Left, fully stochastic damage, e.g. due to exposure to solar UV radiation or tobacco smoke. Center, molecular damage arising from constraint and AP, e.g. reduced end-to-end joining of chromosomes and reduced telomeric DNA repair, due to the shelterin complex (Fumagalli et al., 2012). Right, molecular damage caused by pathology arising from other causes, e.g. oxidation arising from reperfusion damage after stroke.

Considering this relationship allows three sources of molecular damage to be distinguished (Figure 9). First, molecular damage that is not the result of programmatic mechanisms, but is wholly stochastic in origin. This would include damage due to extrinsic stressors such as solar UV radiation or tobacco smoke. Second, molecular damage occurring when pathology leads to major loss of homeostasis, as in anoxia followed by reperfusion damage due to stroke (brain hemorrhage); such damage caused by (rather than causing) senescence is perhaps the main source of molecular damage occurring towards the end of life (Blagosklonny, 2008b). Third, molecular damage arising as a consequence of biological constraint. Some molecular damage can be understood as the consequence of wild-type processes, what Vladimir Dilman described as “regular stochastic processes” (Dilman, 1994).

Regarding constraint: processes exist where it is impossible to set all fitness benefits to optimal, and fitness costs include molecular damage. This may be described as *programmatic molecular damage*. For example, during protein translation it is not possible to maximize both protein translation rate and translation fidelity (Conn and Qian, 2013). Potentially, increasing protein translation rate to support rapid growth may lead to increased levels of protein synthesis errors and protein aggregation (i.e. molecular damage). This would exemplify programmatic molecular damage, arising from constraint, and trade-offs set by genes that would be expected to exhibit AP.

Another example of programmatic molecular damage relates to ROS generated by NADPH oxidases (Nox and Duox enzymes) which serves various functions (e.g. in innate immunity, signal transduction, biochemical reactions) but contributes later to inflammaging and its various associated diseases (Lambeth, 2007).

Another example of programmatic molecular damage that might contribute to aging involves telomeres. Naked DNA at chromosomal ends is prone to end-to-end joining, leading to aneuploidy. This is prevented by the telomeric shelterin complex, including TRF2 (telomeric repeat binding factor 2). However, the presence of this complex also impedes DNA repair at telomeres, leading to increased DNA damage and telomere shortening (Fumagalli et al., 2012). Here an organizational constraint prevents simultaneous optimization of telomeric DNA repair and prevention of aneuploidy.

## 6 Final remarks

The development of programmatic theory has, we believe, helped inch the aging field closer towards attainment of an effective explanatory paradigm in evolutionary physiology terms, something that it has lacked until now (Gems and de Magalhães, 2021). In this essay we have endeavored to build upon foundations created in particular by Williams, Blagosklonny and de Magalhães, to elaborate and extend this paradigm. In particular, we show how consideration of the role of constraint in the evolution of aging helps to make sense of programmatic mechanisms of aging. This creates a broader ultimate proximate account of the causes of aging, that includes an expanding family of programmatic mechanisms and a growing toolbox of new terminology (see Glossary), which we hope will be helpful in enabling new ways of thinking about programmatic aging. We note that some of the concepts described here were developed, in parallel and independently, by Wensink and Cohen (2022). This includes the importance of biological constraint in the evolution of aging, the introduction of a typology similar to interconnection and impossibility constraint, the deduction that constraint leads to the evolution of hyperfunction, and to evolutionary causes of aging distinct from mutation accumulation and antagonistic pleiotropy (Wensink and Cohen, 2022).

Supplementary Table 1 includes diverse examples of spandrels arising from constraint unconnected to known AP genes, and constraints to which neither spandrels or AP genes have been associated, as well as many actual and potential examples of AP. Detailed discussion of selected examples is presented in the Supplementary Discussion. This includes several cases of constraint due to gene action differing between cell types; the manifold constraints arising from multicellularity may explain why programmatic senescence occurs more at the tissue level than the cellular and subcellular levels (Gems, 2022). That constraint arises from proximate mechanisms but shapes the evolutionary process also illustrates the limitations of Ernst Mayr’s ultimate proximate dichotomy (Mayr, 1961; Sinervo and Svensson, 1998). Arguably, ultimate determinants of aging can only be fully understood by taking into consideration the proximate mechanisms that constrain them (Wensink and Cohen, 2022). A further possibility is that differences in the constraints present in different taxa (e.g. phyla) may contribute to evolved variation in aging rate across the tree of life (Wensink and Cohen, 2022). Finally, our account raises one last question, relating to wild-type function as a cause of disease.

### 6.1 Wild-type genes cause disease

As a broad approximation, diseases of aging can be understood as resulting from two principal types of cause (Gems, 2022). First, disruptions of wild-type biological function, e.g. due to infectious pathogens, mechanical injury, mutation, malnutrition etc. Second, and more importantly, the wild-type genome, whose later, pathogenic action is ultimately caused by the process of evolution, including selection shadows and constraint, which gives rise to AP.

This binary scheme includes a distinction between genes that cause disease because they are mutant (disrupted, defective) and wild-type genes that cause disease due to AP. But do all genes that cause disease fall clearly into one of these two categories? Following this argument through to its logical conclusion, let us consider variation at the *Htt* (Huntingtin) locus. Here alleles with higher numbers of CAG repeats, leading to longer polyglutamine tracts, cause Huntington’s disease (HD), a severe and fatal neurodegenerative disease with mid-life onset. *Htt* alleles that cause HD may also increase resistance to cancer (McNulty et al., 2018; Sorenson et al., 1999), perhaps due to increased expression of the pro-apoptotic tumor suppressor protein p53 (Eskenazi et al., 2007)(Supplemental Discussion). Thus *Htt* alleles that cause HD exhibit AP and should therefore be considered to be wild-type, particularly given a history of positive selection (Byars and Voskarides, 2020; Peng et al., 2007). (Though HD has been used to illustrate the mutation accumulation theory (Haldane, 1941) it is, plausibly, an example of AP).

As a second example, consider the F508del allele of *CFTR* (cystic fibrosis transmembrane conductance regulator) which when heterozygous promotes resistance to cholera infection (Gabriel et al., 1994; Meindl, 1987) but when homozygous causes cystic fibrosis. Here, again, F508del is a wild-type allele that is disease-causing due to AP. In this example, instead of providing early-life benefit and promoting late-life disease, as in disease-causing *Htt* alleles, it benefits the heterozygote and causes disease in the homozygote.

While this view of disease-causing *Htt* and *CFTR* alleles might initially seem counterintuitive, it should be remembered that the more common *Htt* alleles increase cancer susceptibility, while those of *CFTR* increase cholera susceptibility: they too are disease-promoting alleles. Moreover, AP at wild-type alleles is potentially the main cause of late-life disease and death.

A plausible assumption is that most AP genes are undetectable, due to absence of alleles with different effects on the antagonistic traits; genes with the latter may represent the tip of the iceberg. Possibly all genes exhibit AP; a recent study comparing allele frequency changes in aging cohorts of outbred *Drosophila* maintained in just two different conditions identified thousands of lifespan-associated allelic variants (Pallares et al., 2023). That AP genes where allelic variation exists often also exhibit overdominance (Supplementary Table 1) is due to the inevitable balancing selection in such cases, as fitness is increased in the heterozygote and decreased in the homozygote. The impossibility of optimizing fitness in both the heterozygote and the homozygote represents a further form of constraint (*allele dosage constraint*).

## Supplementary Material

Supplementary Materials

## Glossary

*Adaptive death*: Synonymous with programmed organismal death. Here death of an individual is a selected trait, providing a direct benefit in terms of inclusive or group fitness (Lohr et al., 2019)
***Allele dosage constraint*** (new term): Where it is not possible to simultaneously optimize the effect of a given allele in both the homozygous and heterozygous state. This gives rise to overdominance (heterozygous advantage)
***Antagonistic pleiotropy*** *(AP)*: Where action of a given gene is both beneficial and detrimental to function, health and/or fitness. If the detriment occurs later in life and is therefore subject to weaker selection, such a gene may be favored by natural selection, and promote aging (Williams, 1957)
***Asynchronous constraint*** (new term): A form of biological constraint where fitness traits that cannot be simultaneously optimized occur at different points in the life history
***Asynchronous trade-off*** (new term): Trade-off where cost and benefit are temporally separated, which can result from either asynchronous or synchronous constraints
***Asynchronous developmental constraint*** (new term): Where a change to a developmental program leading to a trait change necessarily causes an additional later trait change. This is a potential cause of antagonistic pleiotropy acting through programmatic mechanisms (e.g. where the later trait change is quasi-program driven)
***Bad spandrel*** (new term): A trait that evolves as a side effect of selection for another, linked trait, that has deleterious effects on health and/or fitness. Deleterious traits caused by AP genes are bad spandrels
*Biological constraint*: A property of organisms and/or their ecology that prevents the evolution of traits that would increase fitness. This includes selective and organizational constraints. The latter includes impossibility and interconnection constraints
***Clock speed constraint*** (new term): Where changes in overall rate of development and aging occur, the relative timing of reproduction and aging is fixed (constrained). Thus, a more rapid rate of living leads to earlier reproduction (potential fitness benefit) and earlier aging (potential fitness cost)
*Costly program*: A biological program that simultaneously promotes fitness and incurs a cost in terms of pathological changes to tissues or organs where the program is executed. This is one form of programmatic mechanism involving hyperfunction by which AP causes senescence (cf. quasi-program) (Gems et al., 2021)
*Disposable soma theory*: This proposes that natural selection favors investment of limited resources into reproduction rather than somatic maintenance, thereby increasing fitness but accelerating damage accumulation and, therefore, senescence (Kirkwood, 1977)
***Firehose-type costly program*** (new term): Where a function providing protection against an acute, potentially severe threat to health causes collateral injury to tissue, e.g. degranulation by phagocytes. The analogy is with the damage caused by the water from firefighters’ hoses as they extinguish a fire
*Hyperfunction*: Where wild-type gene function and processes actively lead to senescent pathology, as opposed to passive random damage or wear and tear (Blagosklonny, 2006)
*Hypofunction*: Where deficiency in function encoded by the wild-type genome promotes pathology (Maklakov and Chapman, 2019). Hypofunction can result from interconnection constraints or impossibility constraints
***Impossibility constraint*** (new term): Where a function that would provide a fitness benefit is absent due to the impossibility of its evolution. Impossibility constraint gives rise to hypofunction manifesting as phenotypic insufficiency. For example, the human menopause likely exists due to an impossibility constraint having blocked the evolution of capacity to produce viable oocytes over a longer period. Impossibility may result from traits being either *unfeasible* (e.g. production of additional teeth by elderly elephants) or *unreachable* (e.g. after gene loss in a clade, as in uricase in higher primates)
***Interconnection constraint*** (new term): A type of organizational constraint where multiple traits are structurally or functionally interconnected. As a consequence, an evolutionary change affecting one trait leads to changes in one or more additional traits
*Iteroparous*: Possessing the capacity for more than one cycle of reproduction
*Maturo-developmental*: This describes developmental processes, involving e.g. growth, differentiation and morphogenesis, that occur after ontogenesis, i.e. during adulthood. These include both global developmental changes (e.g. post-maturational growth) and more localized changes such as those linked to reproduction, and to diverse processes of tissue repair and immunity (Gems et al., 2024)
***Mixed constraint*** (new term): Where simultaneous maximization of all benefits of a trait is not possible due to constraints on both selective and organizational benefits. This may particularly affect interactions between infectious pathogens and the evolution of immune function (where organizational constraints are operative in the latter)
***Molecular constraint*** (new term): Where organizational constraint exists because a protein possesses more than one biochemical activity, and optimizing all activities at once is not possible. E.g. *AR* (androgen receptor), see Supplementary Discussion
***Multiplex constraint*** (new term): Where organizational constraints affect multiple characteristics. Many genes (e.g. *AAT1* α1-antitrypsin) show several distinct forms of AP involving different mechanisms, reflecting the presence of multiplex constraint
*Organizational constraint*: Where the optimization of biological function is constrained (Acerenza, 2016). This can result either from the highly integrated nature of biological systems, such that improving one aspect causes deterioration of another (interconnection constraint); or because evolution of a given trait is not possible (impossibility constraint)
*Programmed aging*: Senescence caused by a relatively ordered series of biological processes that promotes fitness via inclusive fitness or group fitness. Programmed aging is thought to occur only in certain species, largely those with colonial lifestyles (e.g. colonial microbes) (Galimov and Gems, 2021; Lohr et al., 2019)
*Programmatic aging*: Where complex, wild-type biological processes contributes to senescence, but where senescence does not necessarily contribute to fitness (cf. quasi-programs, costly programs). Programmed aging is a subset of programmatic aging
***Programmatic molecular damage*** (new term): Where molecular damage occurs as a consequence of biological constraint, such that program action increases levels of molecular damage
*Quasi-program*: A cause of senescence entailing a relatively ordered series of biological processes that does not promote fitness; quasi-programs may occur e.g. due to futile run-on of wild-type programs that promote fitness earlier in life (Blagosklonny, 2006), or be triggered later in life (Kern and Stebbing, 2023) (cf. programmatic aging)
*Reproductive death*: A form of suicidal reproductive effort found in some semelparous species (e.g. Pacific salmon, monocarpic plants). Here, reproductive maturity triggers the rapid development of lethal pathologies and fast senescence coupled to reproductive success (Finch, 1990) (chapter 2)
*Run-on*: Futile continuation of gene function or processes in later life, leading to pathology (de la Guardia et al., 2016) (cf. quasi-program)
*Selective constraint*: Where a trait is under opposing forces of selection due to different ecological determinants (Acerenza, 2016). For example, male túngara frogs (*Engystomops pustulosus*) croak in order to attract females, which also attracts a predator, the fringe-lipped bat (*Trachops cirrhosus*) (Tuttle and Ryan, 1981)
*Semelparous*: Organisms with a single reproductive episode before death. Also sometimes used to denote semelparity with reproductive death
*Senescence*: The overall process of deterioration with age or the resulting pathological condition (not to be confused with *cellular senescence* sensu Hayflick, which is a particular form of cellular change affecting some vertebrate cell types) (Gems and Kern, 2022). Although *aging* has several meanings, in the biological context it is usually synonymous with senescence
***Sexual dimorphism constraint*** (new term): Where selection for a trait in one sex leads to its expression in the other. Examples in humans include male nipples and, potentially, post-menopausal longevity in women (Gems, 2014; Gould, 1991)
***Signaling constraint*** (new term): Where a given signaling molecule (e.g. hormone or growth factor, receptor, signaling kinase, transcription factor) acts in diverse contexts (cell types, tissues, organs), such that optimization of function in all contexts is not possible. For example, mTOR integrates diverse stimuli (e.g. growth factors, insulin, nutritional status, stress) and interacts with diverse proteins to control various cellular processes (Kim and Guan, 2019)
*Spandrel*: A trait that evolves as a side effect of selection for another, linked trait (Gould and Lewontin, 1979). Traits originating as spandrels may be beneficial, neutral or harmful in terms of biological function and fitness
***Synchronous constraint*** (new term): A form of biological constraint where the opposing forces of natural selection, or the interconnectedness of traits that give rise to the constraint, exert their effects simultaneously
***Synchronous trade-off*** (new term): A form of trade-off where costs and benefits are experienced simultaneously, that arises from synchronous constraint

## References

[R1] Acerenza L (2016). Constraints, trade-offs and the currency of fitness. J Mol Evol.

[R2] Antonovics J, van Tienderen P (1991). Ontoecogenophyloconstraints? The chaos of constraint terminology. Trends Ecol Evol.

[R3] Arnold KR, Rose MR (2023). Conceptual Breakthroughs in The Evolutionary Biology of Aging.

[R4] Arnold S (1992). Constraints on phenotypic evolution. Am Nat.

[R5] Austad S, Hoffman J (2018). Is antagonistic pleiotropy ubiquitous in aging biology?. Evol Med Public Health.

[R6] Austad SN (1994). Menopause: An evolutionary perspective. Exp Gerontol.

[R7] Bar-Even A, Flamholz A, Noor E, Milo R (2012). Rethinking glycolysis: on the biochemical logic of metabolic pathways. Nat Chem Biol.

[R8] Blagosklonny MV (2006). Aging and immortality: quasi-programmed senescence and its pharmacologic inhibition. Cell Cycle.

[R9] Blagosklonny MV (2007). Paradoxes of aging. Cell Cycle.

[R10] Blagosklonny MV (2008a). Aging, stem cells, and mammalian target of rapamycin: a prospect of pharmacologic rejuvenation of aging stem cells. Rejuvenation Res.

[R11] Blagosklonny MV (2008b). Aging: ROS or TOR. Cell Cycle.

[R12] Blagosklonny MV (2013). TOR-centric view on insulin resistance and diabetic complications: perspective for endocrinologists and gerontologists. Cell Death Dis.

[R13] Brakefield P, Roskam J (2006). Exploring evolutionary constraints is a task for an integrative evolutionary biology. Am Nat.

[R14] Brand MD (2000). Uncoupling to survive? The role of mitochondrial inefficiency in ageing. Exp Geront.

[R15] Brüne M, Schiefenhövel W (2019). The Oxford Handbook of Evolutionary Medicine.

[R16] Butler PG, Wanamaker AD, Scourse JD, Richardson CA, Reynolds DJ (2013). Variability of marine climate on the North Icelandic Shelf in a 1357-year proxy archived based on growth increments in the bivalve *Arctica islandica*. Palaeogeogr Palaeocl.

[R17] Byars SG, Voskarides K (2020). Antagonistic pleiotropy in human disease. J Mol Evol.

[R18] Campisi J (2013). Aging, cellular senescence, and cancer. Annu Rev Physiol.

[R19] Carter AJR, Nguyen AQ (2011). Antagonistic pleiotropy as a widespread mechanism for the maintenance of polymorphic disease alleles. BMC Med Genet.

[R20] Chapman H, Hsiung KC, Rawlinson I, Galimov ER, Gems D (2024). Colony level fitness analysis identifies a trade-off between population growth rate and dauer yield in *Caenorhabditis elegans*. BMC Ecol Evol.

[R21] Conn CS, Qian SB (2013). Nutrient signaling in protein homeostasis: an increase in quantity at the expense of quality. Sci Signal.

[R22] Dahl M, Tybjaerg-Hansen A, Sillesen H, Jensen G, Steffensen R, Nordestgaard BG (2003). Blood pressure, risk of ischemic cerebrovascular and ischemic heart disease, and longevity in alpha(1)-antitrypsin deficiency: the Copenhagen City Heart Study. Circulation.

[R23] de la Guardia Y, Gilliat AF, Hellberg J, Rennert P, Cabreiro F, Gems D (2016). Run-on of germline apoptosis promotes gonad senescence in *C. elegans*. Oncotarget.

[R24] de Magalhães JP (2012). Programmatic features of aging originating in development: aging mechanisms beyond molecular damage?. FASEB J.

[R25] de Magalhães JP, Church GM (2005). Genomes optimize reproduction: aging as a consequence of the developmental program. Physiology.

[R26] Di Rienzo A, Hudson RR (2005). An evolutionary framework for common diseases: the ancestral-susceptibility model. Trends Genet.

[R27] Dilman VM (1994). Development, Aging and Disease: A New Rationale for an Intervention Strategy.

[R28] Endler J (1980). Natural selection of color patterns in Poecilia reticulata. Evolution.

[R29] Eskenazi BR, Wilson-Rich NS, Starks PT (2007). A Darwinian approach to Huntington's disease: subtle health benefits of a neurological disorder. Med Hypotheses.

[R30] Estevez M, Attisano L, Wrana JL, Albert PS, Massague J, Riddle DL (1993). The *daf-4* gene encodes a bone morphogenetic protein receptor controlling *C. elegans* dauer larva development. Nature.

[R31] Finch CE (1990). Longevity, Senescence and the Genome.

[R32] Fumagalli M, Rossiello F, Clerici M, Barozzi S, Cittaro D, Kaplunov JM, Bucci G, Dobreva M, Matti V, Beausejour CM, Herbig U (2012). Telomeric DNA damage is irreparable and causes persistent DNA-damage-response activation. Nat Cell Biol.

[R33] Gabriel S, Brigman K, Koller B, Boucher R, Stutts B (1994). Cystic fibrosis heterozygous resistance to cholera toxin in the cystic fibrosis mouse model. Science.

[R34] Galimov ER, Gems D (2020). Shorter life and reduced fecundity can increase colony fitness in virtual *C. elegans*. Aging Cell.

[R35] Galimov ER, Gems D (2021). Death happy: Adaptive death and its evolution by kin selection in organisms with colonial ecology. Philos Trans R Soc B.

[R36] Galimov ER, Lohr JN, Gems D (2019). When and how can death be an adaptation?. Biochemistry (Moscow).

[R37] Gavrilets S, Rice WR (2006). Genetic models of homosexuality: generating testable predictions. Proc Biol Sci.

[R38] Gems D (2014). Evolution of sexually dimorphic longevity in humans. Aging.

[R39] Gems D (2022). Understanding hyperfunction: an emerging paradigm for the biology of aging. Ageing Res Rev.

[R40] Gems D (2024). How aging causes osteoarthritis: an evolutionary physiology perspective.

[R41] Gems D, de la Guardia Y (2013). Alternative perspectives on aging in C. elegans: reactive oxygen species or hyperfunction?. Antioxid Redox Signal.

[R42] Gems D, de Magalhães JP (2021). The hoverfly and the wasp: A critique of the hallmarks of aging as a paradigm. Ageing Res Rev.

[R43] Gems D, Kern CC (2022). Is “cellular senescence” a misnomer?. Geroscience.

[R44] Gems D, Kern CC, Nour J, Ezcurra M (2021). Reproductive suicide: similar mechanisms of aging in *C. elegans* and Pacific salmon. Front Cell Dev Biol.

[R45] Gems D, Singh Virk R, de Magalhães JP (2024). Epigenetic clocks and programmatic aging. Ageing Res Rev.

[R46] Gould SJ (1980). The evolutionary biology of constraint. Daedalus.

[R47] Gould SJ (1991). Male Nipples and Clitoral Ripples, Bully for Brontosaurus.

[R48] Gould SJ (1997). The exaptive excellence of spandrels as a term and prototype. Proc Natl Acad Sci U S A.

[R49] Gould SJ, Lewontin RC (1979). The spandrels of San Marco and the Panglossian paradigm: a critique of the adaptationist programme. Proc R Soc Lond B.

[R50] Grandison RC, Piper MD, Partridge L (2009). Amino-acid imbalance explains extension of lifespan by dietary restriction in Drosophila. Nature.

[R51] Guillaume F, Otto S (2012). Gene functional trade-offs and the evolution of pleiotropy. Genetics.

[R52] Haldane JBS (1941). New Paths in Genetics.

[R53] Hamilton WD (1966). The moulding of senescence by natural selection. J Theor Biol.

[R54] Hodgkin J (1998). Seven types of pleiotropy. Int J Dev Biol.

[R55] Hong J-Y, Lan T-Y, Tang G-J, Tang C-H, Chen T-J, Lin H-Y (2015). Gout and the risk of dementia: a nationwide population-based cohort study. Arthritis Res Ther.

[R56] Hosoya A, Shalehin N, Takebe H, Shimo T, Irie K (2020). Sonic hedgehog signaling and tooth development. Int J Mol Sci.

[R57] Kern C, Stebbing J (2023). Uncovering the blueprint of aging: how aging causes late-life disease.

[R58] Kim J, Guan K-L (2019). mTOR as a central hub of nutrient signalling and cell growth. Nat Cell Biol.

[R59] King E, Roff D, Fairbairn D (2010). Tradeoff acquisition and allocation in Gryllus firmus: a test of the Y model. J Evol Biol.

[R60] Kirkwood TB (2005). Understanding the odd science of aging. Cell.

[R61] Kirkwood TBL (1977). Evolution of ageing. Nature.

[R62] Kirkwood TBL, Rose MR (1991). Evolution of senescence: late survival sacrificed for reproduction. Phil Trans R Soc London.

[R63] Kratzer JT, Lanaspa MA, Murphy MN, Cicerchi C, Graves CL, Tipton PA, Ortlund EA, Johnson RJ, Gaucher EA (2014). Evolutionary history and metabolic insights of ancient mammalian uricases. Proc Natl Acad Sci U S A.

[R64] Labbadia J, Morimoto R (2015). Repression of the heat shock response is a programmed event at the onset of reproduction Mol. Cell.

[R65] Lambeth JD (2007). Nox enzymes, ROS, and chronic disease: An example of antagonistic pleiotropy. Free Radical Biol Med.

[R66] Lee PC, Sayialel S, Lindsay WK, Moss CJ (2012). African elephant age determination from teeth: validation from known individuals. African J Ecol.

[R67] Lemaître J-F, Berger V, Bonenfant C, Douhard M, Gamelon M, Plard F, Gaillard J-M (2015). Early-late life trade-offs and the evolution of ageing in the wild. Proc Biol Sci.

[R68] Lemaître J-F, Moorad J, Gaillard J-M, Maklakov AA, Nussey DH (2024). From hallmarks of ageing to natural selection: A unified framework for genetic and physiological evolutionary theories of ageing. PLoS Biol.

[R69] Levine DA, Boyd J (2001). The androgen receptor and genetic susceptibility to ovarian cancer: results from a case series. Cancer Res.

[R70] Lewontin R (1947). The Genetic Basis of Evolutionary Change.

[R71] Listì F, Candore G, Grimaldi MP, Lio D, Colonna-Romano G, Orlando V, Caruso M, Hoffmann E, Paolisso G, Franceschi C, Caruso C (2007). Alpha1-antitrypsin heterozygosity plays a positive role in attainment of longevity. Biogerontology.

[R72] Lohr J, Galimov ER, Gems D (2019). Does senescence promote fitness in Caenorhabditis elegans by causing death?. Ageing Res Rev.

[R73] Lu N, Dubreuil M, Zhang Y, Neogi N, Rai SK, Ascherio A, Hernán MA, Choi HK (2016). Gout and the risk of Alzheimer’s disease: a population-based, BMI-matched cohort study. Ann Rheum Dis.

[R74] Maklakov AA, Chapman T (2019). Evolution of ageing as a tangle of trade-offs: energy versus function. Proc Biol Sci.

[R75] Marlowe F (2000). The patriarch hypothesis: An alternative explanation of menopause. Human Nature.

[R76] Mauro AA, Ghalambor CK (2020). Trade-offs, pleiotropy, and shared molecular pathways: a unified view of constraints on adaptation. Integr Comp Biol.

[R77] Maynard Smith J, Burian R, Kauffman S, Alberch P, Campbell J, Goodwin B, Lande R, Raup D, Wolpert L (1985). Developmental constraints and evolution: a perspective from the Mountain Lake Conference on Development and Evolution. Q Rev Biol.

[R78] Maynard Smith J, Haigh J (1974). The hitch-hiking effect of a favourable gene. Genet Res.

[R79] Mayr E (1961). Cause and effect in biology. Science.

[R80] McNulty P, Pilcher R, Ramesh R, Necuiniate R, Hughes A, Farewell D, Holmans P, Jones L, Network, R.I.o.t.E.H.s.D (2018). Reduced cancer incidence in Huntington’s disease: analysis in the registry study. J Huntingtons Dis.

[R81] Medawar PB (1952). An Unsolved Problem Of Biology.

[R82] Meindl R (1987). Hypothesis: A selective advantage for cystic fibrosis heterozygotes. Am J Phys Anthropol.

[R83] Nesse RM, Williams GC (1994). Why We Get Sick: The New Science of Darwinian Medicine.

[R84] Nyström T (2004). Growth versus maintenance: a trade-off dictated by RNA polymerase availability and sigma factor competition?. Mol Microbiol.

[R85] Paaby AB, Rockman MV (2013). The many faces of pleiotropy. Trends Genet.

[R86] Pakpoor J, Seminog OO, Ramagopalan SV, Goldacre MJ (2015). Clinical associations between gout and multiple sclerosis, Parkinson’s disease and motor neuron disease: record-linkage studies. BMC Neurol.

[R87] Pallares LF, Lea AJ, Han C, Filippova EV, Andolfatto P, Ayroles JF (2023). Dietary stress remodels the genetic architecture of lifespan variation in outbred Drosophila. Nat Genet.

[R88] Peng J, Redman CM, Wu X, Song X, Walker RH, Westhoff CM, Lee S (2007). Insights into extensive deletions around the XK locus associated with McLeod phenotype and characterization of two novel cases. Gene.

[R89] Pfeiffer T, Schuster S, Bonhoeffer S (2001). Cooperation and competition in the evolution of ATP-producing pathways. Science.

[R90] Piper M, Soultoukis G, Blanc E, Mesaros A, Herbert S, Juricic P, He X, Atanassov I, Salmonowicz H, Yang M, Simpson S (2017). Matching dietary amino acid balance to the in silico-translated exome optimizes growth and reproduction without cost to lifespan. Cell Metab.

[R91] Predazzi IM, Rokas A, Deinard A, Schnetz-Boutaud N, Williams ND, Bush WS, Tacconelli A, Friedrich K, Fazio S, Novelli G, Haines JL (2013). Putting pleiotropy and selection into context defines a new paradigm for interpreting genetic data. Circ Cardiovasc Genet.

[R92] Promislow DEL (2004). Protein networks, pleiotropy and the evolution of senescence. Proc R Soc Lond B.

[R93] Raj K, Horvath S (2020). Current perspectives on the cellular and molecular features of epigenetic ageing. Exp Biol Med.

[R94] Rebbeck TP, Kantoff PW, Krithivas K, Neuhausen S, Blackwood MA, Godwin AK, Daly MB, Narod SA, Garber JE, Lynch HT, Weber BL (1999). Modification of BRCA1-associated breast cancer risk by the polymorphic androgen-receptor CAG repeat. Am J Hum Genet.

[R95] Robson SL, Wood B (2008). Hominin life history: reconstruction and evolution. J Anat.

[R96] Rook GAW, Brüne M, Schiefenhövel W (2019). The Oxford Handbook of Evolutionary Medicine.

[R97] Sapey E, Greenwood H, Walton G, Mann E, Love A, Aaronson N, Insall RH, Stockley RA, Lord JM (2014). Phosphoinositide 3-kinase inhibition restores neutrophil accuracy in the elderly: toward targeted treatments for immunosenescence. Blood.

[R98] Sinervo B, Svensson E (1998). Mechanistic and selective causes of life history trade-offs and plasticity. Oikos.

[R99] Slade L, Etheridge T, Szewczyk NL (2024). Consolidating multiple evolutionary theories of ageing suggests a need for new approaches to study genetic contributions to ageing decline. Ageing Res Rev.

[R100] Sorenson SA, Fenger K, Olsen J (1999). Significantly lower incidence of cancer among patients with Huntington’s disease. Cancer.

[R101] Speakman JR (2008). The physiological costs of reproduction in small mammals. Philos Trans R Soc Lond B Biol Sci.

[R102] Speakman JR, Król E (2010). The heat dissipation limit theory and evolution of life histories in endotherms--time to dispose of the disposable soma theory?. Integr Comp Biol.

[R103] Strenk SA, Strenk LM, Koretz JF (2005). The mechanism of presbyopia. Prog Retin Eye Res.

[R104] Suvorov A (2022). Modalities of aging in organisms with different strategies of resource allocation. Ageing Res Rev.

[R105] Teulière J, Bernard C, Corel E, Lapointe FJ, Martens J, Lopez P, Bapteste E (2023). Network analyses unveil ageing-associated pathways evolutionarily conserved from fungi to animals. Geroscience.

[R106] Tuttle MD, Ryan MJ (1981). Bat predation and the evolution of frog vocalizations in the neotropics. Science.

[R107] van Exel E, Koopman JJE, von Bodegom D, Meij JJ, de Knijff P, Ziem JB, Finch CE, Westendorp RGJ (2017). Effect of APOE ε4 allele on survival and fertility in an adverse environment. PLoS ONE.

[R108] van Noordwijk AJ, de Jong G (1986). Acquisition and allocation of resources: their influence on variation in life history tactics. Am Nat.

[R109] Voskarides K (2018). Combination of 247 genome-wide association studies reveals high cancer risk as a result of evolutionary adaptation. Mol Biol Evol.

[R110] Voskarides K (2019). The "cancer-cold" hypothesis and possible extensions for the Nordic populations. Scand J Public Health.

[R111] Voynow JA, Shinbashi M (2021). Neutrophil elastase and chronic lung disease. Biomolecules.

[R112] Wang H, Zhao Y, Ezcurra M, Benedetto A, Gilliat A, Hellberg J, Ren Z, Athigapanich T, Girstmair J, Telford MJ, Dolphin CT (2018). A parthenogenetic quasi-program causes teratoma-like tumors during aging in wild-type C. elegans. NPJ Aging Mech Disease.

[R113] Weisskopf MG, O’Reilly E, Chen H, Schwarzschild MA, Ascherio A (2007). Plasma urate and risk of Parkinson’s disease. Am J Epidemiol.

[R114] Wensink MJ, Cohen AA (2022). The Danaid Theory of Aging. Front Cell Dev Biol.

[R115] Westendorp R (2015). Growing Older Without Feeling Old.

[R116] Williams GC (1957). Pleiotropy, natural selection and the evolution of senescence. Evolution.

[R117] Williams GC (1992). Domains, Levels and Challenges.

[R118] Zajitschek F, Georgolopoulos G, Vourlou A, Ericsson M, Zajitschek S, Friberg U, Maklakov A (2019). Evolution under dietary restriction decouples survival from fecundity in Drosophila melanogaster females. J Gerontol A Biol Sci Med Sci.

[R119] Zera AJ, Harshman LG (2001). The physiology of life history trade-offs in animals. Annu Rev Ecol Syst.

[R120] Zhang B, Gems D (2021). Gross ways to live long: Parasitic worms as an anti-inflammaging therapy?. eLife.

[R121] Zhao X, Promislow DEL, Brüne M, Schiefenhövel W (2019). The Oxford Handbook of Evolutionary Medicine.

[R122] Zhao Y, Gilliat AF, Ziehm M, Turmaine M, Wang H, Ezcurra M, Yang C, Phillips G, McBay D, Zhang WB, Partridge L (2017). Two forms of death in aging Caenorhabditis elegans. Nat Commun.

