# Supplementary material for: Biological constraint, evolutionary spandrels and antagonistic pleiotropy
Source: Ageing Res Rev. Author manuscript; Available in PMC 2026 Jan 6. (PMC7618566; doi:10.1016/j.arr.2024.102527)
Supplement: Supplementary Materials [file EMS211721-suppement-Supplementary_Materials.pdf]

Supplementary Table 1: 100+ examples of antagonistic pleiotropy, spandrels and biological constraint

Example[1]	Possible type of constraint	Possible pathogenetic mechanism	Sources	Notes[2]
**Antagonistic pleiotropy, spandrels**				
AAT1 α1-antitrypsin inhibits neutrophil elastase, aiding migration of neutrophils through tissue, which supports immunity but causes tissue damage (e.g. destruction of elastin) that promotes COPD.	Mixed (including interconnection)	Costly program, firehose type, hyperfunction.	(Sapey et al., 2014; Stoller and Aboussouan, 2012; Voynow and Shinbashi, 2021)	Elastin in extracellular matrix destroyed by neutrophil elastase is not replaced.
AAT1 Z allele protects against myocardial infarction (heterozygote) and promotes longevity, but may increase risk of COPD.	Interconnection	The M ("wild-type") allele is hypofunctional with respect to correcting hypertension.	(Dahl et al., 2003; Listì et al., 2007; Tanash et al., 2020)	Elastase also reduces hypertension, perhaps by breaking down elastic tissue in the arterial wall and altering vessel distensibility. This reduces myocardial infarction.
AAT1 Z allele protects against myocardial infarction (heterozygote) and promotes longevity, but may increase risk of COPD.	Interconnection	The M allele C-36 promotes an atherogenic quasi-program, hyperfunction	(Dahl et al., 2003; Dichtl et al., 2000; Tanash et al., 2020)	Cleavage of AAT generates the C-36 peptide which stimulates inflammatory processes that promote atherogenesis.
ADRB2 beta-2 adrenergic receptor *Arg16Gly, Gln27Glu* polymorphisms improve early life cognition but increase late-life disease (hypertension, myocardial infarction, cancer).	Interconnection	Unclear. Constraint on diverse functions of ADRB2 leads to hyperfunction or hypofunction.	(Bao et al., 2005; Bochdanovits et al., 2009; Cagliani et al., 2009; Kulminski et al., 2010)	ADRB2 plays multiple roles in different organs and tissues, making interpretation of AP difficult.
ALOX15 G SNP increases bone mineral density before menopause but promotes osteoporosis after menopause (femoral neck).	Interconnection, asynchronous developmental?	Run-on and/or triggered quasi-program, hyperfunction.	(Cheung et al., 2008)	ALOX15 is arachidonate 15-lipoxygenase.
APOE4 allele associated with improved cognition when there is a high parasite burden, but increases risk of Alzheimer's disease and cardiovascular disease.	Interconnection	Unclear.	(Trumble et al., 2017)	Evidence from Amazonian forager-horticulturalists (Tsimane people, Bolivia).
APOE4 allele confers female reproductive advantage, but increases risk of Alzheimer's disease and cardiovascular disease.	Interconnection	Unclear.	(Corbo et al., 2004; Jasienska et al., 2015; van Exel et al., 2017)	*APOE4* allele increases luteal progesterone, increasing fertility (Jasienska et al., 2015).
APOE4 allele increases aspects of cognitive performance in early life, but increases risk of Alzheimer's disease and cardiovascular disease.	Interconnection	Unclear.	(Alexander et al., 2007; Jochemsen et al., 2012; Rusted et al., 2013; Wright et al., 2003; Yu et al., 2000)	*APOE4* allele improves performance in tests of memory (including visual working memory in later life)(Lu et al., 2021), attention, verbal fluency, and mental arithmetic.
APOE4 allele protects against effects of childhood infections with Giardia and cryptosporidium, but increases risk of Alzheimer's disease and cardiovascular disease.	Interconnection	Unclear.	(Oriá et al., 2007; Oriá et al., 2010; Oriá et al., 2005)	Evidence from Brazilian children living in poverty, and experiencing diarrhea.
APOE4 allele may protect against hepatitis C, but increases risk of Alzheimer's disease and cardiovascular disease.	Interconnection	Unclear.	(Mueller et al., 2016)	
AR (androgen receptor) variants from alleles with shorter CAG repeat length have higher androgen sensitivity. In young men, this increases reproductive fitness (increased sperm viability, enhanced traits attractive to females), but in later life increases prostate cancer.	Interconnection, asynchronous developmental?	Run-on and/or triggered quasi-program, leading to benign prostatic hyperplasia, hyperfunction.	(Butovskaya et al., 2015; Dowsing et al., 1999; Ingles et al., 1997)	
AR variants from alleles with longer CAG repeat length reduce prostate cancer, but longer polyglutamine tracts can cause neurodegeneration (Kennedy's disease).	Interconnection, molecular constraint	Programmatic molecular damage.	(La Spada et al., 1991)	

AR variants from alleles with shorter CAG repeat length reduce risk of breast cancer, but increase risk of ovarian cancer.	Interconnection, asynchronous developmental?	Run-on and/or triggered quasi-program, hyperfunction.	(Levine and Boyd, 2001; Rebbeck et al., 1999)	
AR with shorter CAG repeat length increases reproductive fitness in men but increases ovarian cancer in women.	Interconnection, sexual dimorphism constraint, asynchronous developmental?	Run-on and/or triggered quasi-program, hyperfunction.	(Levine and Boyd, 2001; Rebbeck et al., 1999)	
BRCA1/2 mutation can increase female fertility under natural fertility conditions, and ovarian cancer and breast cancer.	Interconnection	Run-on or triggered quasi-program, hyperfunction (hypofunction).	(Smith et al., 2012)	Loss of a tumor suppressor gene leading to hyperproliferation is hypofunction leading to hyperfunction.
BRCA1/2 mutation promotes breast development, but also ovarian and breast cancer.	Interconnection, asynchronous developmental?	Run-on and/or triggered quasi-program, hyperfunction (hypofunction).	(Crespi and Summers, 2006)	Tentative.
CFTR mutation increases fertility (heterozygote) but causes cystic fibrosis (homozygote).	Allele dosage	Hypofunction.	(Knudson et al., 1967)	Trans-membrane chloride channel defect. Overdominance.
CFTR mutation increases cholera resistance (heterozygote) but causes cystic fibrosis (homozygote).	Mixed (including allele dosage)	Hypofunction.	(Gabriel et al., 1994; Meindl, 1987)	Suggested by findings with mice, and human cell culture.
CFTR mutation increases resistance resistance to influenza and other myxo- and paramyxoviruses (heterozygote) but causes cystic fibrosis (homozygote).	Mixed (including allele dosage)	Hypofunction.	(Shier, 1979)	Suggested by cell culture work.
FRM1 mutation increases female fertility (heterozygote) but causes mental retardation in hemizygous males (fragile X syndrome).	Allele dosage	Hypofunction.	(Vogel et al., 1990)	Overdominance.
G6PD mutation protects against malaria (heterozygote) but causes hemolytic anemia (homozygote/hemizygote).	Mixed (including allele dosage)	Hypofunction.	(Clark et al., 2009; Guindo et al., 2007)	Glucose-6-phosphate dehydrogenase deficiency.
GHR (growth hormone receptor) mutation increases resistance to cancer, but causes Laron syndrome (including dwarfism).	Interconnection	Hypofunction.	(Guevara-Aguirre et al., 2011)	Wild-type GHR is hyperfunctional in later life, contributing to cancer.
GROW1/GDF1 variant promotes joint and bone development and osteoarthritis.	Interconnection, asynchronous developmental?	Run-on and/or triggered quasi-program, hyperfunction.	(Capellini et al., 2017)	
HBB β-globin mutation protects against falciparum malaria (heterozygote), but causes sickle cell anemia (homozygote).	Mixed (including allele dosage)	Hypofunction.		Overdominance. Wild-type HBB is hyperfunctional insofar as it increases susceptibility to malaria.
HBB mutation protects against malaria (heterozygote) but causes β-thalassemia (homozygote).	Mixed (including allele dosage)	Hypofunction.	(Haldane, 1949)	Overdominance.
HEXA mutation may increase resistance to tuberculosis, and does cause Tay Sachs disease.	Mixed	Hypofunction.	(Diamond, 1988)	HEXA encodes the alpha-subunit of the lysosomal enzyme beta-N-acetylhexosaminidase. Tentative.
HFE (high Fe^{2+} protein) mutation improves dietary iron uptake and protects against typhoid fever and tuberculosis (heterozygote) but causes hemachromatosis, including liver damage (homozygote).	Mixed (including allele dosage)	Hyperfunction.	(Weinberg, 2008)	Overdominance.
Htt (Huntingtin). High CAG number *Htt* alleles increase fertility but cause Huntington's disease.	Interconnection, asynchronous developmental?	Run-on and/or triggered quasi-program, hyperfunction.	(Shokeir, 1975; Walker et al., 1983)	Increased fertility may be due to altered behavior, reflecting cognitive changes.

Htt. High CAG number *Htt* alleles reduce incidence of various forms of cancer but cause Huntington's disease.	Interconnection, asynchronous developmental?	Run-on and/or triggered quasi-program, hyperfunction.	(Eskenazi et al., 2007; McNulty et al., 2018; Sorenson et al., 1999)	High CAG number *Htt* alleles increase apoptosis, perhaps by increasing levels of the pro-apoptotic and anti-cancer protein p53. This may contribute to increased neuronal apoptosis, and neurodegeneration.
ORL1 (lectin-like low-density lipoprotein receptor 1) promotes immune defense by binding bacterial cell wall proteins, but also promotes atherosclerosis and cardiovascular disease by binding oxidized low density lipoprotein in endothelial cells.	Mixed	Quasi-program, hyperfunction.	(Predazzi et al., 2013)	
PAH (phenylalanine hydroxylase) mutation provides protection against mold-induced miscarriage (heterozygote), but causes phenylketonuria (homozygote).	Mixed (including allele dosage)	Hypofunction.	(Woolf, 1986)	Resistance to ochratoxin A, an N-acyl derivative of phenylalanine, and mycotoxin produced by some *Aspergillus* and *Penicillium* species. Overdominance. Tentative.
PTPN11 mutation protects against leukemia but increases hepatocellular carcinoma (mouse).	Interconnection	Quasi-program, hyperfunction.	(Bard-Chapeau et al., 2011)	*PTPN11* (proto-oncogene) encodes the shp2 protein.
TNFRSF11B expression at higher levels increases bone mineral density but also risk of several epithelial cancers, and affects tumor angiogenesis.	Interconnection	Quasi-program, hyperfunction.	(Ito et al., 2003; Samelson et al., 2008)	*TNFRSF11B* encodes the cytokine receptor protein osteoprogesterin.
SPATA31 higher copy number improves sensing of UV-induced DNA damage and sun damage resistance but increases cellular senescence and, perhaps, aging.	Interconnection	Hyperfunction?	(Bekpen et al., 2018)	Spermatogenesis-associated protein 31.
TP53 (p53) variants affect female fertility, longevity and cancer risk.	Interconnection	Hyperfunction (hypofunction).	(Kang et al., 2009; Olivier et al., 2010; Ørsted et al., 2007)	Loss of a tumor suppressor gene leading to hyperproliferation is hypofunction leading to hyperfunction.
Multiple loci protect against Crohn's disease and ulcerative colitis but promote type 1 diabetes.	Interconnection	Unknown	(Wang et al., 2010)	
Positive selection drives clonal expansion of cells with tumor suppressor mutations (*TP53, NOTCH1*) among healthy oesophageal cells due to increased resistance to stomach acidity.	Interconnection	Hyperfunction (hypofunction).	(Martincorena et al., 2018; Yokoyama et al., 2019)	Loss of a tumor suppressor gene leading to hyperproliferation is hypofunction leading to hyperfunction.
Uricase loss protects against neurodegeneration (vascular dementia, Alzheimer's disease, Parkinson's disease) but causes gout (especially in older men).	Interconnection	Hypofunction, leading to programmatic molecular damage.	(Hong et al., 2015; Lu et al., 2016; Pakpoor et al., 2015; Weisskopf et al., 2007)	Most mammals possess uricase, but it has been lost among higher primates, possibly to protect brain longevity.
Chromosomal regions 6p22-p24 and 11q21-22 increase fertility but increase risk of schizophrenia.	Interconnection	Unknown.	(Srinivasan and Padmavati, 1997)	
Tumor suppressor mutations provide benefits in extreme cold and at high altitudes (e.g. by reducing apoptosis) but increase cancer risk (e.g. Inuit, Tibetans, Scandinavians).	Interconnection	Quasi-program, hyperfunction.	(Voskarides, 2018; Voskarides, 2019)	Cancer-cold hypothesis.
GHR mutation increases lifespan but causes dwarfism (mouse).	Interconnection	Hypofunction.	(Coschigano et al., 2000)	*GHR*, growth hormone receptor.
Prop1 mutation increases lifespan but causes combined pituitary hormone deficiency (mouse).	Interconnection	Hypofunction.	(Brown-Borg et al., 1996)	*Prop1*, prophet of *pit-1*, transcription factor promoting anterior pituitary development.
Region of chromosome 17 specifies negative correlation between dimensions of two bones in the skull (in the zygomatic arch) due to developmental constraint (mouse).	Interconnection, synchronous developmental	No clear fitness cost.	(Percival et al., 2018)	
oca2 mutation increases brain norepinephrine levels but causes albinism (*Astyanax mexicanus* fish).	Interconnection	(Hypofunction). Within caves, albinism is not a fitness cost.	(Bilandžija et al., 2018)	Mexican tetra (blind cave fish). Loss of melanin synthesis increases tyrosine

				availability for catecholamine synthesis (e.g. norepinephrine).
Xmrk oncogene increases melanin pigmentation pattern promoting male mating success, but causes melanoma in males (*Xiphophorus cortezi* fish).	Mixed, selective (sexual selection) and organizational	Run-on type quasi-program, hyperfunction.	(Fernandez and Bowser, 2010; Fernandez and Morris, 2008)	*Xmrk* (*Xiphophorus* melanoma receptor kinase), constitutively active form of epidermal growth factor receptor causing melanocyte hyperproliferation and melanoma.
Xmrk increases male size but causes melanoma in males (*X. cortezi*).	Selective: S. vs N. (sexual selection vs natural selection)	Run-on type quasi-program, hyperfunction.	(Fernandez and Bowser, 2010; Fernandez and Morris, 2008)	
Xmrk increases melanin pattern promoting male mating success, but causes melanoma in females (*X. cortezi*).	Interconnection, sexual dimorphism	Run-on type quasi-program, hyperfunction.	(Fernandez and Bowser, 2010; Fernandez and Morris, 2008)	
Xmrk causes more melanoma in homozygotes than heterozygotes (*X. cortezi*).	Interconnection, gene dosage	Run-on type quasi-program, hyperfunction.	(Fernandez and Bowser, 2010; Fernandez and Morris, 2008)	
daf-2 mutation strongly increases lifespan but increases larval diapause thereby reducing fertility, and reduces population growth and survival under some conditions (*C. elegans*).	Interconnection, asynchronous developmental	*daf-2(+)* hypofunction, with respect to aging.	(Jenkins et al., 2004; Riddle et al., 1981; Van Voorhies et al., 2005)	
tra-3(e2333) mutation increases brood size by extending spermatogenesis but reduces fitness by delaying onset of fertilization (*C. elegans*).	Interconnection, asynchronous developmental	*tra-3(+)* hyperfunction, leading to hypofunction with respect to brood size.	(Hodgkin and Barnes, 1991)	Protandry in *C. elegans* enables rapid reproduction and resource colonization but causes early cessation of reproduction.
RPM1 polymorphism increases plant pathogen resistance but reduces growth rate (*Arabidopsis thaliana*).	Mixed	Hypofunction.	(Tian et al., 2003)	*RPM1*, Resistance to *Pseudomonas syringae pv. maculicola* 1.
Chromosome rearrangement can increase mitotic rates but impair sexual reproduction (meiosis) (*Schizosaccharomyces pombe*).	Interconnection	Hypofunction.	(Avelar et al., 2013)	
Loss of *rpoS*, encoding σ^S subunit of RNA polymerase, increases growth under nutrient-replete conditions, but reduces resistance to starvation (*Escherichia coli*).	Interconnection	Hypofunction.	(Nyström, 2004)	Different σ factors compete for access to limited amounts of RNA polymerase holoenzyme, creating trade-offs between them.
**Spandrels**				
The chin.	Architectural constraint	None.	(Gould and Lewontin, 1979; Williams, 1992)	The chin appears to be a futile product of human jaw evolution, left over after the more massive dental arcades of our hominin ancestors shrank to their modern size.
Dihydrotestosterone promotes seminal fluid production by the prostate gland throughout adulthood, but promotes benign prostatic hyperplasia, and prostate cancer in later life (human, chimpanzee).	Interconnection, synchronous developmental	Quasi-program, hyperfunction.	(Untergasser et al., 2005)	
Evolutionary change leading to improved human cognitive function has been linked to autistic spectrum disorder and schizophrenia.	Interconnection	Unknown.	(Duński and Pękowska, 2022)	Comparisons of the genomes of humans and chimpanzees, Denisovans and Neanderthals show recent changes in genes linked to brain function that are also linked to the named disorders.
Gay, same-sex sexual behavior/orientation linked to increased sexual activity, fecundity in heterosexual relatives.	Interconnection, sexual dimorphism	None.	(Camperio-Ciani et al., 2004; King et al., 2005; Zietsch et al., 2021)	Selected, genetic determinants of increased sexual activity in heterosexuals may promote homosexual orientation in some individuals.

Genes altering joint morphology to facilitate bipedalism in later life have been suggested to promote osteoarthritis.	Interconnection, asynchronous, developmental	Unknown.	(Aubourg et al., 2022)	
IGF-1 at high levels in youth promote health but in late life increase risk of mortality, dementia, vascular disease, diabetes, osteoporosis, and cancer.	Interconnection, asynchronous, developmental?	Run-on and/or triggered quasi-program, hyperfunction.	(Zhang et al., 2021)	
Presbyopia (long-sightedness due to loss of eye lens elasticity).	None	Run-on quasi-program, hyperfunction.	(Strenk et al., 2005)	Late life run-on of lens growth leads to increase in lens thickness, impairing vision.
TGFβ1 in seminal fluid protects sperm within the female by suppressing female immunity, but in the male causes fibroblast to myofibroblast trans-differentiation within the prostate, leading to epithelial cell hypertrophy (male humans).	Interconnection, asynchronous developmental?	Run-on and/or triggered quasi-program, hyperfunction.	(Untergasser et al., 2005)	
The clitoris (female mammals).	Interconnection, sexual dimorphism	None.	(Gould, 1991)	Potentially a spandrel, arising from the male penile development program expressed in females (cf male nipples).
Male nipples (mammals).	Interconnection, sexual dimorphism	None.	(Gould, 1991)	A spandrel arising from the female breast development program expressed in females (cf clitoris).
Female prostate (Skene's gland), female ejaculation (mammals)	Interconnection, sexual dimorphism	None.	(Toivanen and Shen, 2017)	A likely spandrel, arising from the male prostate development program expressed in females.
In male babirusas (a species of wild pig) tusk-like protruding canines point backwards over the snout, whose continued growth can pierce the cranium (genus *Babyrousa*).	None	Run-on quasi-program, hyperfunction.	(Macdonald, 2018)	As a run-on quasi-program, this is an example of a constraint-independent spandrel.
Pandas have evolved a functional false thumb from the radial sesamoid bone of the wrist. There is also a non-adaptive expansion of the equivalent bone in the foot (the tibial sesamoid).	Interconnection, synchronous, developmental	None.	(Gould, 1992)	Evolution appears to have acted on mechanisms affecting equivalent growth fields in hand and foot development.
Synaptic pruning promotes neurodegeneration (mammals).	None	Run-on quasi-program, hyperfunction.	(De Magalhaes and Sandberg, 2005)	Synaptic pruning in the brain promotes cognitive development, but then runs on in later life, leading to age-related cognitive decline (hypothetical).
mTOR promotes cell growth but in cells that have exited the cell cycle, promotes geroconversion (hypertrophic and hypersecretory states of senescent cells) (mammals).	Interconnection, asynchronous developmental?	Run-on and/or triggered quasi-program, hyperfunction.	(Blagosklonny, 2014)	
mTOR promotes growth but in later life promotes multiple diseases of aging (mammals).	Interconnection, asynchronous developmental?	Run-on and/or triggered quasi-program, hyperfunction.	(Blagosklonny, 2008b; Tsang et al., 2007)	E.g. cardiomyocyte hypertrophy contributing to cardiac hypertrophy; hypertrophy and hypersecretion in "senescent" cells.
Snail umbilicus.	Architectural constraint.	None.	(Gould, 1997)	Snails that grow by coiling a tube around a central axis generate a cylindrical space, the umbilicus. A few species use this as a brooding chamber to protect the eggs (exaptation).
**Biological constraints**				
Aβ functions as an antimicrobial but promotes Alzheimer's disease.	Mixed, interconnection,	Run-on and/or triggered quasi-program, hyperfunction.	(Moir and Tanzi, 2019; Soscia et al., 2010)	

	asynchronous developmental?			
Aβ regulates cholesterol transport but promotes Alzheimer's disease.	Interconnection, asynchronous developmental?	Run-on and/or triggered quasi-program, hyperfunction.	(Igbavboa et al., 2009; Yao and Papadopoulos, 2002)	
Aβ (β amyloid) provides protection against oxidative stress but promotes Alzheimer's disease.	Interconnection, asynchronous developmental?	Run-on and/or triggered quasi-program, hyperfunction.	(Baruch-Suchodolsky and Fischer, 2009; Zou et al., 2002)	
Aβ functions as a transcription factor but promotes Alzheimer's disease.	Interconnection, asynchronous developmental?	Run-on and/or triggered quasi-program, hyperfunction.	(Bailey et al., 2011; Maloney and Lahiri, 2011)	
Angiotensin II receptor promotes organ development and function, and also cardiovascular disease (e.g. hypertension, atherosclerosis, cardiac hypertrophy), heart failure, diabetes, chronic kidney disease, dementia, osteoporosis, and cancer.	Interconnection, asynchronous developmental?	Run-on and/or triggered quasi-program, hyperfunction.	(Kamo et al., 2015)	
Autism is associated with improvements in some aspects of cognitive function.	Interconnection	Hypofunction?	(Crespi, 2016)	
Bipolar disorder is associated with increased creativity.	Interconnection	Costly program?	(Redfield Jamison, 1993)	
Bone breakdown by osteoclasts during lactation releases Ca^{2+} for milk production but promotes osteoporosis, especially after menopause.	Interconnection, asynchronous developmental	Costly program, triggered quasi-program, hyperfunction.	(Speakman, 2008)	
General resistance to cell death may protect against Alzheimer's disease but increase cancer risk.	Interconnection, asynchronous developmental?	Run-on and/or triggered quasi-program, hyperfunction.	(Li et al., 2014; Staropoli, 2008)	Individuals with AD have reduced cancer risk and vice versa (Ma et al., 2014; Shi et al., 2015; Zhang et al., 2015).
Cellular senescence is a tumor suppressor mechanism but senescent cell accumulation promotes cancer.	Interconnection, asynchronous developmental?	Run-on and/or triggered quasi-program, hyperfunction.	(Campisi, 1997)	
High levels of C-reactive protein, a marker of inflammation (immune defense), is associated with memory impairment in non-demented elderly.	Mixed	Costly program/quasi-program, hyperfunction.	(Silverman et al., 2009)	
Diarrhea may help clear intestinal infections (e.g. *Shigella*, a cause of dysentery) but chronic diarrhea can cause dehydration and infant death.	Mixed	Costly program, firehose type, hyperfunction.	(DuPont and Hornick, 1973; Tsai et al., 2017)	Tentative.
Fever protects against infection, but is temporarily disabling and can cause febrile seizures in children.	Mixed	Costly program, firehose type, hyperfunction.	(Nesse and Williams, 1994)	Effects of fever are largely benign.
Fibroblast "senescence" promotes wound healing but SASP promotes multiple diseases of aging.	Interconnection, asynchronous developmental?	Run-on and/or triggered quasi-program, hyperfunction.	(Demaria et al., 2014)	
Gastric acidity protects against infection but increases risk of gastric and duodenal ulcers.	Mixed	Costly program, firehose type, hyperfunction.	(Albin, 1988; Rotter and Diamond, 1987)	
Immune responses protect against pathogens, but can cause autoimmune diseases (e.g. lupus erythematosus, rheumatoid arthritis).	Mixed	Costly program, firehose type/quasi-program, hyperfunction.		
Inflammatory responses to bacterial infection help clear infection but if over-induced (e.g. during sepsis) the resulting cytokine storm can cause fatal immune suppression.	Mixed	Costly program, firehose type/quasi-program, hyperfunction.	(Nedeva et al., 2019)	

Innate immunity protects against infection but can increase miscarriage rate by causing rejection of fetus.	Mixed	Costly program/quasi-program, hyperfunction.	(Van Bodegom et al., 2007)	
Elevated insulin reduces hyperglycemia but promotes diabetic retinopathy by increasing neovascularization.	Interconnection, asynchronous developmental?	Run-on and/or triggered quasi-program, hyperfunction.	(Blagosklonny, 2013)	Insulin stimulates mTOR, which activates VEGF and angiogenesis.
Low iron protects against bacterial and protozoan pathogens (e.g. malaria) and cancer, but causes anemia.	Mixed	Hypofunction.	(Weinberg, 1984)	
Absence of iterogametogenesis due to gametogenic program design leads to exhaustion of oocyte stocks, menopause and loss of fertility.	Impossibility, unreachable	Hypofunction.	(Austad, 1994)	
NADPH oxidases (Nox, Duox enzymes) generate ROS for diverse functions (innate immunity, signal transduction, biochemical reactions) which also contributes to inflammaging, and resultant diseases .	Mixed, interconnection	Costly program, firehose type/quasi-program, hyperfunction. Programmatic molecular damage.	(Lambeth, 2007)	
Pain perception conditions avoidance of noxious stimuli, but untreatable, chronic illness can produce futile chronic pain.	Interconnection	Costly program, firehose type/quasi-program, hyperfunction.		
Aggregation and adhesion of platelets promotes blood clotting and wound healing, but also thrombosis (e.g. stroke, myocardial infarction, pulmonary embolism).	Interconnection, asynchronous developmental?	Run-on and/or triggered quasi-program, hyperfunction.	(Blagosklonny, 2006)	
The shelterin complex prevents telomere end joining and aneuploidy, but impedes telomeric DNA repair, increasing DNA damage and telomere shortening.	Interconnection	Hypofunction, programmatic molecular damage (as it is a consequence of constraint).	(Fumagalli et al., 2012)	
Skin pigmentation protects against solar radiation injury but also reduces vitamin D synthesis.	Interconnection	Hypofunction.		
mTOR reduces cellular overgrowth by promoting signal resistance in growth factor receptors, also causing insulin resistance, and age-related loss of stem cell function.	Interconnection, asynchronous developmental?	Run-on and/or triggered quasi-program, hyperfunction.	(Blagosklonny, 2006; Blagosklonny, 2008a)	
Cessation of successional tooth development (elephant).	Impossibility, unreachable	Hypofunction	(Lee et al., 2012) (This study)	
Wnt/β-catenin signaling promotes normal development but also muscle stem cell exhaustion during aging (mouse).	Interconnection, asynchronous developmental?	Run-on and/or triggered quasi-program, hyperfunction.	(Brack et al., 2007; Naito et al., 2012)	Wnt hyperfunction promotes switch from myogenic to fibrogenic lineage.
Males are brightly colored to attract females, which also attracts predators (guppy, *Poecilia reticulata*).	Selection	Predation.	(Endler, 1980)	The predator here is the pike cichlid (*Crenicichla alta*).
Males croak to attract females, which also attracts predators (túngara frog, *Engystomops pustulosus*).	Selection	Predation.	(Tuttle and Ryan, 1981)	The predator here is the fringe-lipped bat (*Trachops cirrhosus*).
Sex peptides in seminal fluid increase egg production but reduce female lifespan (*Drosophila melanogaster*).	Interconnection	Costly program?	(Wigby and Chapman, 2005)	
Phenoloxidase protects against infection but causes tissue damage in later life (mealworm beetle *Tenebrio molitor*).	Interconnection	Firehose-type costly program.	(Khan et al., 2017)	Immune effector causes immunopathological costs by damaging Malpighian tubules (equivalent to mammalian kidney).
Loss of the flagellum during cell division (unicellular algae).	Mixed	Predation.	(Michod, 2007)	The function of the flagellum requires the centriole. The latter is lost during cell division, increasing vulnerability to predation. This creates a trade-off between survival (aided by flagellar locomotion) and reproduction.

Increasing ATP production rate reduces yield and vice versa (general biology).	Interconnection	Hyperfunction, hypofunction.	(Pfeiffer et al., 2001)	
Increasing enzyme specificity can reduce speed and vice versa (general biology).	Interconnection	Hyperfunction, hypofunction.	(Tawfik, 2014)	
Protein occupancy constraint generates trade-offs between reproductive and stress resistance functions (general biology, particularly bacteria).	Interconnection	Hypofunction, programmatic molecular damage.	(Acerenza, 2016)	Constraint on space for proteins promoting growth (e.g. ribosomal) and survival (e.g. molecular chaperones) leads to trade-offs between growth and stress resistance.

For AP examples, mutation, spandrel and constraint are described. For spandrel examples, only spandrel and constraint are described. For constraint examples, constraint alone is described. For some examples, operative constraints and pathogenetic mechanisms are suggested hypotheses to varying degrees. The many examples presented provide opportunities for further investigation, and a testing ground for the ideas framework presented in this report.

[1]Human, unless otherwise stated.
[2]Listed examples of categories of phenomena are selected either on the basis of their interpretation as such in cited sources, or our interpretation of them as such. In some cases, further work is required to verify the proposed occurrence of AP.

Supplemental Table 1 references

Acerenza, L., 2016. Constraints, trade-offs and the currency of fitness. J. Mol. Evol. 82, 117–127.

Albin, R.L., 1988. The pleiotropic gene theory of senescence: supportive evidence from human genetic disease. Ethol. Sociobiol. 9, 371-382.

Alexander, D.M., Williams, L.M., Gatt, J.M., Dobson-Stone, C., Kuan, S.A., Todd, E.G., Schofield, P.R., Cooper, N.J. and Gordon, E., 2007. The contribution of apolipoprotein E alleles on cognitive performance and dynamic neural activity over six decades. Biol. Psychol. 75, 229-238.

Aubourg, G., Rice, S.J., Bruce-Wootton, P. and Loughlin, J., 2022. Genetics of osteoarthritis. Osteoarthritis Cartilage. 30, 636-649.

Austad, S.N., 1994. Menopause: An evolutionary perspective. Exp. Gerontol. 29, 255-263.

Avelar, A.T., Perfeito, L., Gordo, I. and Ferreira, M.G., 2013. Genome architecture is a selectable trait that can be maintained by antagonistic pleiotropy. Nat. Commun. 4, 2235.

Bailey, J.A., Maloney, B., Ge, Y.-W. and Lahiri, D.K., 2011. Functional activity of the novel Alzheimer's amyloid β-peptide interacting domain (AβID) in the APP and BACE1 promoter sequences and implications in activating apoptotic genes and in amyloidogenesis. Gene. 488, 13-22.

Bao, X., Mills, P.J., Rana, B.K., Dimsdale, D.E., Schork, N.J., Smith, D.W., Rao, F., Milic, M., O'Connor, D.T. and Ziegler, M.G., 2005. Interactive effects of common beta2-adrenoceptor haplotypes and age on susceptibility to hypertension and receptor function. Hypertension. 46, 301-307.

Bard-Chapeau, E.A., Li, S., Ding, J., Zhang, S.S., Zhu, H.H., Princen, F., Fang, D.D., Han, T., Bailly-Maitre, B., Poli, V., Varki, N.M., Wang, H. and Feng, G.S., 2011. Ptpn11/Shp2 acts as a tumor suppressor in hepatocellular carcinogenesis. Cancer Cell. 19, 629-639.

Baruch-Suchodolsky, R. and Fischer, B., 2009. Abeta40, either soluble or aggregated, is a remarkably potent antioxidant in cell-free oxidative systems. Biochemistry. 48, 4354-4370.

Bekpen, C., Xie, C., Nebel, A. and Tautz, D., 2018. Involvement of SPATA31 copy number variable genes in human lifespan. Aging (Albany NY). 10, 674-688.

Bilandžija, H., Abraham, L., Ma, L., Renner, K.J. and Jeffery, W.R., 2018. Behavioural changes controlled by catecholaminergic systems explain recurrent loss of pigmentation in cavefish. Proc. Biol. Sci. 285, 20180243.

Blagosklonny, M.V., 2006. Aging and immortality: quasi-programmed senescence and its pharmacologic inhibition. Cell Cycle. 5, 2087-102.

Blagosklonny, M.V., 2008a. Aging, stem cells, and mammalian target of rapamycin: a prospect of pharmacologic rejuvenation of aging stem cells. Rejuvenation Res. 11, 801-8.

Blagosklonny, M.V., 2008b. Aging: ROS or TOR. Cell Cycle. 7, 3344-54.

Blagosklonny, M.V., 2013. TOR-centric view on insulin resistance and diabetic complications: perspective for endocrinologists and gerontologists. Cell Death Dis. 4, e964.

Blagosklonny, M.V., 2014. Geroconversion: irreversible step to cellular senescence. Cell Cycle. 13, 3628-3635.

Bochdanovits, Z., Gosso, F.M., van den Berg, L., Rizzu, P., Polderman, T.J.C., Pardo, L.M., Houlihan, L.M., Luciano, M., Starr, J.M., Harris, S.E., Deary, I.J., de Geus, E.J.C., Boomsma, D.I., Heutink, P. and Posthuma, D., 2009. A Functional polymorphism under positive evolutionary selection in ADRB2 is associated with human intelligence with opposite effects in the young and the elderly. Behav. Genet. 39, 15-23.

Brack, A.S., Conboy, M.J., Roy, S., Lee, M., Kuo, C.J., Keller, C. and Rando, T.A., 2007. Increased Wnt signaling during aging alters muscle stem cell fate and increases fibrosis. Science. 317, 807-810.
Brown-Borg, H.M., Borg, K.E., Meliska, C.J. and Bartke, A., 1996. Dwarf mice and the ageing process. Nature. 384, 33.
Butovskaya, M.L., Lazebny, O.E., Vasilyev, V.A., Dronova, D.A., Karelin, D.V., Mabulla, A.Z.P., Shibalev, D.V., Shackelford, T.K., Fink, B. and Ryskov, A.P., 2015. Androgen receptor gene polymorphism, aggression, and reproduction in Tanzanian foragers and pastoralists. PLoS One. 10, e0136208.
Cagliani, R., Fumagalli, M., Pozzoli, U., Riva, S., Comi, G.P., Torri, F., Macciardi, F., Bresolin, N. and Sironi, M., 2009. Diverse evolutionary histories for beta-adrenoreceptor genes in humans. Am. J. Hum. Genet. 85, 64-75.
Camperio-Ciani, A.C., Corna, F. and Capiluppi, C., 2004. Evidence for maternally inherited factors favouring male homosexuality and promoting female fecundity. Proc. Biol. Sci. 271, 2217-2221.
Campisi, J., 1997. Aging and cancer: the double-edged sword of replicative senescence. J. Am. Geriatr. Soc. 45, 482-488.
Capellini, T.D., Chen, H., Cao, J., Doxey, A.C., Kiapour, A.M., Schoor, M. and Kingsley, D.M., 2017. Ancient selection for derived alleles at a GDF5 enhancer influencing human growth and osteoarthritis risk. Nat. Genet. 49, 1202-1210.
Cheung, C.L., Chan, V. and Kung, A.W.C., 2008. A differential association of ALOX15 Polymorphisms with bone mineral density in pre- and post-menopausal women. Human Heredity. 1, 1-8.
Clark, T.G., Fry, A., Auburn, S., Campino, S., Diakite, M., Green, A., Richardson, A., Teo, Y., Small, K., Wilson, J., Jallow, M., Sisay-Joof, F., Pinder, M., Sabeti, P. and Kwiatkows, D., 2009. Allelic heterogeneity of G6PD deficiency in West Africa and severe malaria susceptibility. Eur. J. Hum. Genet. 17, 1080-1085.
Corbo, R.M., Ulizzi, L., Scacchi, R., Martinez-Labarga, C. and De Stefano, G.F., 2004. Apolipoprotein E polymorphism and fertility: a study in pre-industrial populations Mol. Hum. Reprod. 10, 617-620.
Coschigano, K.T., Clemmons, D., Bellush, L.L. and Kopchick, J.J., 2000. Assessment of growth parameters and life span of GHR/BP gene-disrupted mice. Endocrinology. 141, 2608-2613.
Crespi, B. and Summers, K., 2006. Positive selection in the evolution of cancer. Biol. Rev. Camb. Philos. Soc. 81, 407-424.
Crespi, B.J., 2016. Autism as a disorder of high intelligence. Front. Neurosci. 10, 300.
Dahl, M., Tybjaerg-Hansen, A., Sillesen, H., Jensen, G., Steffensen, R. and Nordestgaard, B.G., 2003. Blood pressure, risk of ischemic cerebrovascular and ischemic heart disease, and longevity in alpha(1)-antitrypsin deficiency: the Copenhagen City Heart Study. Circulation. 107, 747-752.
De Magalhaes, J. and Sandberg, A., 2005. Cognitive aging as an extension of brain development: a model linking learning, brain plasticity, and neurodegeneration. Mech. Ageing Dev. 126, 1026-1033.
Demaria, M., Ohtani, N., Youssef, S., Rodier, F., Toussaint, W., Mitchell, J., Laberge, R., Vijg, J., Van Steeg, H., Dollé, M., Hoeijmakers, J., de Bruin, A., Hara, E. and Campisi, J., 2014. An essential role for senescent cells in optimal wound healing through secretion of PDGF-AA. Dev. Cell. 31, 722-733.
Diamond, J., 1988. Tay-Sachs carriers and tuberculosis resistance. Nature. 331, 666-667.
Dichtl, W., Moraga, F., Ares, M.P., Crisby, M., Nilsson, J., Lindgren, S. and Janciauskiene, S., 2000. The carboxyl-terminal fragment of alpha1-antitrypsin is present in atherosclerotic plaques and regulates inflammatory transcription factors in primary human monocytes. Mol. Cell. Biol. Res. Commun. 4, 50-61.

Dowsing, A.T., Yong, E.L., Clark, M., McLachlan, R.I., de Kretser, D.M. and Trounson, A.O., 1999. Linkage between male infertility and trinucleotide repeat expansion in the androgen-receptor gene. Lancet. 354, 640-643.

Duński, E. and Pękowska, A., 2022. Keeping the balance: Trade-offs between human brain evolution, autism, and schizophrenia. Front. Genet. 13, 1009390.

DuPont, H.L. and Hornick, R.B., 1973. Adverse effect of lomotil therapy in shigellosis. JAMA. 226, 1525-1528.

Endler, J., 1980. Natural selection of color patterns in Poecilia reticulata. Evolution. 34, 76–91.

Eskenazi, B.R., Wilson-Rich, N.S. and Starks, P.T., 2007. A Darwinian approach to Huntington's disease: subtle health benefits of a neurological disorder. Med. Hypotheses. 69, 1183-1189.

Fernandez, A. and Bowser, P., 2010. Selection for a dominant oncogene and large male size as a risk factor for melanoma in the Xiphophorus animal model. Mol. Ecol. 19, 3114-3123.

Fernandez, A.A. and Morris, M.R., 2008. Mate choice for more melanin as a mechanism to maintain a functional oncogene. Proc. Natl. Acad. Sci. U S A. 105, 13503-13507.

Fumagalli, M., Rossiello, F., Clerici, M., Barozzi, S., Cittaro, D., Kaplunov, J.M., Bucci, G., Dobreva, M., Matti, V., Beausejour, C.M., Herbig, U., Longhese, M.P. and d'Adda di Fagagna, F., 2012. Telomeric DNA damage is irreparable and causes persistent DNA-damage-response activation. Nat. Cell Biol. 14, 355-365.

Gabriel, S., Brigman, K., Koller, B., Boucher, R. and Stutts, B., 1994. Cystic fibrosis heterozygous resistance to cholera toxin in the cystic fibrosis mouse model. Science. 266, 107-110.

Gould, S.J., 1991. Male Nipples and Clitoral Ripples, Bully for Brontosaurus. Penguin, London, pp. 124-138.

Gould, S.J., 1992. The Panda's Thumb: More Reflections in Natural History, Norton, New York.

Gould, S.J., 1997. The exaptive excellence of spandrels as a term and prototype. Proc. Natl. Acad. Sci. U S A. 94, 10750-10755.

Gould, S.J. and Lewontin, R.C., 1979. The spandrels of San Marco and the Panglossian paradigm: a critique of the adaptationist programme. Proc. R. Soc. Lond. B. 205, 581-598.

Guevara-Aguirre, J., Balasubramanian, P., Guevara-Aguirre, M., Wei, M., Madia, F., Cheng, C., Hwang, D., Martin-Montalvo, A., Saavedra, J., Ingles, S., de Cabo, R., Cohen, P. and Longo, V., 2011. Growth hormone receptor deficiency is associated with a major reduction in pro-aging signaling, cancer, and diabetes in humans. Sci. Transl. Med. 3, 70ra13.

Guindo, A., Fairhurst, R.M., Doumbo, O.K., Wellens, T.E. and Diallo, D.A., 2007. X-linked G6PD deficiency protects hemizygous males but not heterozygous females against severe malaria. PLoS Med. 4, 0516-0522.

Haldane, J.B.S., 1949. The rate of mutation of human genes. Hereditas. 35, 367-273.

Hodgkin, J. and Barnes, T.M., 1991. More is not better: brood size and population growth in a self-fertilizing nematode. Proc. R. Soc. Lond. B. 246, 19-24.

Hong, J.-Y., Lan, T.-Y., Tang, G.-J., Tang, C.-H., Chen, T.-J. and Lin, H.-Y., 2015. Gout and the risk of dementia: a nationwide population-based cohort study. Arthritis Res. Ther. 17, 139.

Igbavboa, U., Sun, G.Y., Weisman, G.A., He, Y. and Wood, W.G., 2009. Amyloid beta-protein stimulates trafficking of cholesterol and caveolin-1 from the plasma membrane to the Golgi complex in mouse primary astrocytes. Neuroscience. 162, 328-338.

Ingles, S.A., Ross, R.K., Yu, M.C., Irvine, R.A., La Pera, G., Haile, R.W. and Coetzee, G.A., 1997. Association of prostate cancer risk with genetic polymorphisms in vitamin D receptor and androgen receptor. J. Natl. Cancer Inst. 89, 166-170.

Ito, R., Nakayama, H., Yoshida, K., Kuraoka, K., Motoshita, J., Oda, N., Oue, N. and Yasui, W., 2003. Expression of osteoprotegerin correlates with aggressiveness and poor prognosis of gastric carcinoma. Virchows Arch. 443, 146-151.
Jasienska, G., Ellison, P.T., Galbarczyk, A., Jasienski, M., Kalemba-Drozdz, M., Kapiszewska, M., Nenko, I., Thune, I. and Ziomkiewicz, A., 2015. Apolipoprotein E (ApoE) polymorphism is related to differences in potential fertility in women: a case of antagonistic pleiotropy? Proc. Biol. Sci. 282, 20142395.
Jenkins, N.L., McColl, G. and Lithgow, G.J., 2004. Fitness cost of extended lifespan in *Caenorhabditis elegans*. Proc. R. Soc. Lond. B Biol. Sci. 271, 2523-6.
Jochemsen, H.M., Muller, M., van der Graaf, Y. and Geerlings, M.I., 2012. APOE epsilon4 differentially influences change in memory performance depending on age. The SMART-MR study. Neurobiol. Aging. 33, 832.e15-22.
Kamo, T., Akazawa, H. and Komuro, I., 2015. Pleiotropic effects of angiotensin II receptor signaling in cardiovascular homeostasis and aging. Int. Heart J. 56, 249-254.
Kang, H.-J., Feng, Z., Sun, Y., Atwal, G., Murphy, M.E., Rebbeck, T.R., Rosenwaks, Z., Levine, A.J. and Hu, W., 2009. Single-nucleotide polymorphisms in the p53 pathway regulate fertility in humans. Proc. Natl. Acad. Sci. U S A. 106, 9761-9766.
Khan, I., Agashe, D. and Rolff, J., 2017. Early-life inflammation, immune response and ageing. Proc. Biol. Sci. 284.
King, M., Green, J., Osborn, D.P.J., Arkell, J., Hetherton, J. and Pereira, E., 2005. Family size in white gay and heterosexual men. Arch. Sex. Behav. 34, 117-122.
Knudson, A.G., Wayne, L. and Hallett, W.Y., 1967. On selective advantage of cystic fibrosis heterozygotes. Am. J. Hum. Genet. 19, 388-392.
Kulminski, A.M., Culminskaya, I., Ukraintseva, S.V., Arbeev, K.G., Land, K.C. and Yashin, A.I., 2010. Beta2-adrenergic receptor gene polymorphisms as systemic determinants of healthy aging in an evolutionary context. Mech. Ageing Dev. 131, 338-345.
La Spada, A.R., Wilson, E.M., Lubahn, D.B., Harding, A.E. and Fischbeck, K.H., 1991. Androgen receptor gene mutations in X-linked spinal and bulbar muscular atrophy. Nature. 352, 77-79.
Lambeth, J.D., 2007. Nox enzymes, ROS, and chronic disease: An example of antagonistic pleiotropy. Free Radical Biol. Med. 43, 332-347.
Lee, P.C., Sayialel, S., Lindsay, W.K. and Moss, C.J., 2012. African elephant age determination from teeth: validation from known individuals. African J. Ecol. 50, 9-20.
Levine, D.A. and Boyd, J., 2001. The androgen receptor and genetic susceptibility to ovarian cancer: results from a case series. Cancer Res. 61, 908-911.
Li, J.M., Liu, C., Hu, X., Cai, Y., Ma, C., Luo, X.G. and Yan, X.X., 2014. Inverse correlation between Alzheimer's disease and cancer: implication for a strong impact of regenerative propensity on neurodegeneration? BMC Neurol. 14, 211.
Listì, F., Candore, G., Grimaldi, M.P., Lio, D., Colonna-Romano, G., Orlando, V., Caruso, M., Hoffmann, E., Paolisso, G., Franceschi, C. and Caruso, C., 2007. Alpha1-antitrypsin heterozygosity plays a positive role in attainment of longevity. Biogerontology. 8, 139-145.
Lu, K., Nicholas, J.M., Pertzov, Y., Grogan, J., Husain, M., Pavisic, I.M., James, S.-N., Parker, T.D., Lane, C.A., Keshavan, A., Keuss, S.E., Buchanan, S.M., Murray-Smith, H., Cash, D.M., Malone, I.B., Sudre, C.H., Coath, W., Wong, A., Henley, S.M.D., Fox, N.C., Richards, M., Schott, J.M. and Crutch, S.J., 2021. Dissociable effects of APOE-ε4 and β-amyloid pathology on visual working memory. Nat. Aging. 1, 1002-1009.
Lu, N., Dubreuil, M., Zhang, Y., Neogi, N., Rai, S.K., Ascherio, A., Hernán, M.A. and Choi, H.K., 2016. Gout and the risk of Alzheimer's disease: a population-based, BMI-matched cohort study. Ann. Rheum. Dis. 75, 547–551.

Ma, L.L., Yu, J.T., Wang, H.F., Meng, X.F., Tan, C.C., Wang, C. and Tan, L., 2014. Association between cancer and Alzheimer's disease: systematic review and meta-analysis. J. Alzheimers Dis. 42, 565-573.
Macdonald, A.A., 2018. Aberrant growth of maxillary canine teeth in male babirusa (genus *Babyrousa*) Comptes Rendus Biologies. 341, 245-255.
Maloney, B. and Lahiri, D.K., 2011. The Alzheimer's amyloid beta-peptide (Abeta) binds a specific DNA Abeta-interacting domain (AbetaID) in the APP, BACE1, and APOE promoters in a sequence-specific manner: characterizing a new regulatory motif. Gene. 488, 1-12.
Martincorena, I., Fowler, J.C., Wabik, A., Lawson, A.R.J., Abascal, F., Hall, M.W.J., Cagan, A., Murai, K., Mahbubani, K., Stratton, M.R., Fitzgerald, R.C., Handford, P.A., Campbell, P.J., Saeb-Parsy, K. and Jones, P.H., 2018. Somatic mutant clones colonize the human esophagus with age. Science. 362, 911-917.
McNulty, P., Pilcher, R., Ramesh, R., Necuiniate, R., Hughes, A., Farewell, D., Holmans, P., Jones, L. and Network, REGISTRY Investigators of the European Huntington's Disease Network, 2018. Reduced cancer incidence in Huntington's disease: analysis in the registry study. J. Huntingtons Dis. 7, 209-222.
Meindl, R., 1987. Hypothesis: A selective advantage for cystic fibrosis heterozygotes. Am. J. Phys. Anthropol. 74, 39-45.
Michod, R.E., 2007. Evolution of individuality during the transition from unicellular to multicellular life. Proc. Natl. Acad. Sci. U S A. 104, 8613-8618.
Moir, R.D. and Tanzi, R.E., 2019. Low evolutionary selection pressure in senescence does not explain the persistence of Aβ in the vertebrate genome. Front. Aging Neurosci. 11, 70.
Mueller, T., Fischer, J., Gessner, R., Rosendahl, J., Böhm, S., van Bömmel, F., Knop, V., Sarrazin, C., Witt, H., Marques, A.M., Kovacs, P., Schleinitz, D., Stumvoll, M., Blüher, M., Bugert, P., Schott, E. and Berg, T., 2016. Apolipoprotein E allele frequencies in chronic and self-limited hepatitis C suggest a protective effect of APOE4 in the course of hepatitis C virus infection. Liver Int. 36, 1267-74.
Naito, A.T., Sumida, T., Nomura, S., Liu, M.L., Higo, T., Nakagawa, A., Okada, K., Sakai, T., Hashimoto, A., Hara, Y., Shimizu, I., Zhu, W., Toko, H., Katada, A., Akazawa, H., Oka, T., Lee, J.-K., Minamino, T., Nagai, T., Walsh, K., Kikuchi, A., Matsumoto, M., Botto, M., Shiojima, I. and Komuro, I., 2012. Complement C1q activates canonical Wnt signaling and promotes aging-related phenotypes. Cell. 149, 1298–1313.
Nedeva, C., Menassa, J. and Puthalakath, H., 2019. Sepsis: inflammation is a necessary evil. Front. Cell Dev. Biol. 7, 108.
Nesse, R.M. and Williams, G.C., 1994. Why We Get Sick: The New Science of Darwinian Medicine, Random House.
Nyström, T., 2004. Growth versus maintenance: a trade-off dictated by RNA polymerase availability and sigma factor competition? Mol. Microbiol. 54, 855-862.
Olivier, M., Hollstein, M. and Hainaut, P., 2010. TP53 mutations in human cancers: origins, consequences, and clinical use. Cold Spring Harb. Perspect. Biol. 2, a001008.
Oriá, R.B., Patrick, P.D., Blackman, J.A., Lima, A.A. and Guerrant, R.L., 2007. Role of apolipoprotein E4 in protecting children against early childhood diarrhea outcomes and implications for later development. Med. Hypoth. 68, 1099-107.
Oriá, R.B., Patrick, P.D., Oriá, M.O., Lorntz, B., Thompson, M.R., Azevedo, O.G., Lobo, R.N., Pinkerton, R.F., Guerrant, R.L. and Lima, A.A., 2010. ApoE polymorphisms and diarrheal outcomes in Brazilian shanty town children. Braz. J. Med. Biol. Res. 43, 249-56.
Oriá, R.B., Patrick, P.D., Zhang, H., Lorntz, B., de Castro Costa, C.M., Brito, G.A., Barrett, L.J., Lima, A.A. and Guerrant, R.L., 2005. APOE4 protects the cognitive development in children with heavy diarrhea burdens in Northeast Brazil. Pediatr Res. 57, 310-6.

Ørsted, D.D., Bojesen, S.E., Tybjaerg-Hansen, A. and Nordestgaard, B.G., 2007. Tumor suppressor p53 Arg72Pro polymorphism and longevity, cancer survival, and risk of cancer in the general population. J. Exp. Med. 204, 1295-1301.

Pakpoor, J., Seminog, O.O., Ramagopalan, S.V. and Goldacre, M.J., 2015. Clinical associations between gout and multiple sclerosis, Parkinson's disease and motor neuron disease: record-linkage studies. BMC Neurol. 1, 16.

Percival, C.J., Green, R., Roseman, C.C., Gatti, D.M., Morgan, J.L., Murray, S.A., Donahue, L.R., Mayeux, J.M., Pollard, K.M., Hua, K., Pomp, D., Marcucio, R. and Hallgrímsson, B., 2018. Developmental constraint through negative pleiotropy in the zygomatic arch. Evodevo. 9, 3.

Pfeiffer, T., Schuster, S. and Bonhoeffer, S., 2001. Cooperation and competition in the evolution of ATP-producing pathways. Science. 292, 504–507.

Predazzi, I.M., Rokas, A., Deinard, A., Schnetz-Boutaud, N., Williams, N.D., Bush, W.S., Tacconelli, A., Friedrich, K., Fazio, S., Novelli, G., Haines, J.L., Sirugo, G. and Williams, S.M., 2013. Putting pleiotropy and selection into context defines a new paradigm for interpreting genetic data. Circ. Cardiovasc. Genet. 6, 299-307.

Rebbeck, T.P., Kantoff, P.W., Krithivas, K., Neuhausen, S., Blackwood, M.A., Godwin, A.K., Daly, M.B., Narod, S.A., Garber, J.E., Lynch, H.T., Weber, B.L. and Brown, M., 1999. Modification of BRCA1-associated breast cancer risk by the polymorphic androgen-receptor CAG repeat. Am. J. Hum. Genet. 64, 1371-1377.

Redfield Jamison, K., 1993. Touched With Fire: Manic-Depressive Illness and the Artistic Temperament, Simon & Schuster.

Riddle, D.L., Swanson, M.M. and Albert, P.S., 1981. Interacting genes in nematode dauer larva formation. Nature. 290, 668-671.

Rotter, J.I. and Diamond, J.M., 1987. What maintains the frequencies of human genetic diseases? Nature. 329, 289-290.

Rusted, J.M., Evans, S.L., King, S.L., Dowell, N., Tabet, N. and Tofts, P.S., 2013. APOE e4 polymorphism in young adults is associated with improved attention and indexed by distinct neural signatures. Neuroimage. 65, 364-373.

Samelson, E.J., Broe, K.E., Demissie, S., Beck, T.J., Karasik, D., Kathirsean, S. and Kiel, D.P., 2008. Increased plasma osteoprotegerin concentrations are associated with indices of bone strength of the hip. Endocrine Metab. 93, 1789-1795.

Sapey, E., Greenwood, H., Walton, G., Mann, E., Love, A., Aaronson, N., Insall, R.H., Stockley, R.A. and Lord, J.M., 2014. Phosphoinositide 3-kinase inhibition restores neutrophil accuracy in the elderly: toward targeted treatments for immunosenescence. Blood. 123, 239-248.

Shi, H.B., Tang, B., Liu, Y.W., Wang, X.F. and Chen, G.J., 2015. Alzheimer disease and cancer risk: a meta-analysis. J. Cancer Res. Clin. Oncol. 141, 485-494.

Shier, W.T., 1979. Increased resistance to influenza as a possible source of heterozygote advantage in cystic fibrosis. Med. Hypoth. 5, 661-668.

Shokeir, M., 1975. Investigation on Huntington's disease in the Canadian Prairies. II. Fecundity and fitness. Clin. Genet. 7, 349-353.

Silverman, J.M., Schnaider Beeri, M.S., Schmeidler, J., Rosendorff, C., Angelo, G., Mavris, R.S., Grossman, H.T., Elder, G.A., Carrion-Baralt, J. and West, R., 2009. C-reactive protein and memory function suggest antagonistic pleiotropy in very old nondemented subjects. Age Ageing. 38, 237-241.

Smith, K.R., Hanson, H.A., Mineau, G.P. and Buys, S.S., 2012. Effects of BRCA1 and BRCA2 mutations on female fertility. Proc. R. Soc. Lond. B. 279, 1389–1395.

Sorenson, S.A., Fenger, K. and Olsen, J., 1999. Significantly lower incidence of cancer among patients with Huntington's disease. Cancer. 6, 1342-1346.

Soscia, S.J., Kirby, J.E., Washicosky, K.J., Tucker, S.M., Ingelsson, M., Hyman, B., Burton, M.A., Goldstein, L.E., Duong, S., Tanzi, R.E. and Moir, R.D., 2010. The Alzheimer's

disease-associated amyloid beta-protein is an antimicrobial peptide. PLoS One. 5, e9505.
Speakman, J.R., 2008. The physiological costs of reproduction in small mammals. Philos. Trans. R. Soc. Lond. B Biol. Sci. 363, 375-98.
Srinivasan, T. and Padmavati, R., 1997. Fertility and Schizophrenia: Evidence for increased fertility in the relatives of schizophrenic patients. Acta Psychiatric Scand. 96, 260-264.
Staropoli, J.F., 2008. Tumorigenesis and neurodegeneration: two sides of the same coin? Bioessays. 30, 719-727.
Stoller, J.K. and Aboussouan, L.S., 2012. A review of α1-antitrypsin deficiency. Am. J. Respir. Crit. Care Med. 185, 246-259.
Strenk, S.A., Strenk, L.M. and Koretz, J.F., 2005. The mechanism of presbyopia. Prog. Retin. Eye Res. 24, 379-393.
Tanash, H., Ekström, M., Basil, N., Rönmark, E., Lindberg, A. and Piitulainen, E., 2020. Decreased risk of ischemic heart disease in individuals with severe alpha 1-antitrypsin deficiency (PiZZ) in comparison with the general population. Int. J. Chron. Obstruct. Pulmon. Dis. 15, 1245-1252.
Tawfik, D.S., 2014. Accuracy-rate tradeoffs: how do enzymes meet demands of selectivity and catalytic efficiency? Curr. Opin. Chem. Biol. 21, 73-80.
Tian, D., Traw, M.B., Chen, J.Q., Kreitman, M. and Bergelson, J., 2003. Fitness costs of R-gene-mediated resistance in *Arabidopsis thaliana*. Nature. 423, 74-77.
Toivanen, R. and Shen, M.M., 2017. Prostate organogenesis: tissue induction, hormonal regulation and cell type specification. Development. 144, 1382-1398.
Trumble, B.C., Stieglitz, J., Blackwell, A.D., Allayee, H., Beheim, B., Finch, C.E., Gurven, M. and Kaplan, H., 2017. Apolipoprotein E4 is associated with improved cognitive function in Amazonian forager-horticulturalists with a high parasite burden. FASEB J. 31, 1508-1515.
Tsai, P.-Y., Zhang, B., He, W.-Q., Zha, J.-M., Odenwald, M.A., Singh, G., Tamura, A., Shen, L., Sailer, A., Yeruva, S., Kuo, W.-T., Fu, Y.-X., Tsukita, S. and Turner, J.R., 2017. IL-22 upregulates epithelial claudin-2 to drive diarrhea and enteric pathogen clearance. Cell Host Microbe. 21, 671-681.
Tsang, C.K., Qi, H., Liu, L.F. and Zheng, X.F., 2007. Targeting mammalian target of rapamycin (mTOR) for health and diseases. Drug Discov. Today. 12, 112-24.
Tuttle, M.D. and Ryan, M.J., 1981. Bat predation and the evolution of frog vocalizations in the neotropics. Science. 214, 677-678.
Untergasser, G., Madersbacher, S. and Berger, P., 2005. Benign prostatic hyperplasia: age-related tissue-remodeling. Exp. Gerontol. 40, 121-128.
Van Bodegom, D., May, L., Meij, H.J. and Westendorp, R.G.J., 2007. Regulation of human life histories: the role of the inflammatory host response. Ann. N.Y. Acad. Sci. 1100, 84-97.
van Exel, E., Koopman, J.J.E., von Bodegom, D., Meij, J.J., de Knijff, P., Ziem, J.B., Finch, C.E. and Westendorp, R.G.J., 2017. Effect of APOE ε4 allele on survival and fertility in an adverse environment. PLoS ONE. 12, e0179497.
Van Voorhies, W.A., Fuchs, J. and Thomas, S., 2005. The longevity of *Caenorhabditis elegans* in soil. Biol. Lett. 1, 247-249.
Vogel, F., Crusio, W.E., Kovac, C., Fryns, J.P. and Freund, M., 1990. Selective advantage of fra (X) heterozygotes. Hum. Genet. 86, 25-32.
Voskarides, K., 2018. Combination of 247 genome-wide association studies reveals high cancer risk as a result of evolutionary adaptation. Mol. Biol. Evol. 35, 473-485.
Voskarides, K., 2019. The "cancer-cold" hypothesis and possible extensions for the Nordic populations. Scand. J. Public Health. 47, 477-481.
Voynow, J.A. and Shinbashi, M., 2021. Neutrophil elastase and chronic lung disease. Biomolecules. 11, 1065.

Walker, D.A., Harper, P.S., Newcombe, R.G. and Davies, K., 1983. Huntington's chorea in South Wales: mutation, fertility, and genetic fitness. J. Med. Genet. 20, 12-17.
Wang, K., Baldassano, R., Zhang, H., Qu, H.-Q., Imielinski, M., Kugathasan, S., Annese, V., Dubinsky, M., Rotter, J.I., Russell, R.K., Bradfield, J.P., Sleiman, P.M.A., Glessner, J.T., Walters, T., Hou, C., Kim, C., Frackelton, E.C., Garris, M., Doran, J., Romano, C., Catassi, C., Van Limbergen, J., Guthery, S.L., Denson, L., Piccoli, D., Silverberg, M.S., Stanley, C.A., Monos, D., Wilson, D.C., Griffiths, A., Grant, S.F.A., Satsangi, J., Polychronakos, C. and Hakonarson, H., 2010. Comparative genetic analysis of inflammatory bowel disease and type 1 diabetes implicates multiple loci with opposite effects. Hum. Mol. Genet. 19, 2059-2067.
Weinberg, E., 2008. Survival advantage of the hemochromatosis C282Y mutation. Perspect. Biol. Med. 51, 98-102.
Weinberg, E.D., 1984. Iron withholding: a defense against infection and neoplasia. Physiol. Rev. 64, 65-102.
Weisskopf, M.G., O'Reilly, E., Chen, H., Schwarzschild, M.A. and Ascherio, A., 2007. Plasma urate and risk of Parkinson's disease. Am. J. Epidemiol. 166, 561–567.
Wigby, S. and Chapman, T., 2005. Sex peptide causes mating costs in female *Drosophila melanogaster*. Curr. Biol. 15, 316-321.
Williams, G.C., 1992. Historicity and constraint, Natural Selection. Domains, Levels and Challenges. Oxford University Press, New York, pp. 72-88.
Woolf, L.I., 1986. The heterozygote advantage in phenylketonuria. Am. J. Hum. Genet.. 5, 773-775.
Wright, R.O., Hu, H., Silverman, E.K., Tsaih, S.W., Schwartz, J., Bellinger, D., Palazuelos, E., Weiss, S.T. and Hernandez-Avila, M., 2003. Apolipoprotein E genotype predicts 24-month bayley scales infant development score. Pediatr. Res. 54, 819-825.
Yao, Z.-X. and Papadopoulos, V., 2002. Function of beta-amyloid in cholesterol transport: a lead to neurotoxicity. FASEB J. 16, 1677-1679.
Yokoyama, A., Kakiuchi, N., Yoshizato, T., Nannya, Y., Suzuki, H., Takeuchi, Y., Shiozawa, Y., Sato, Y., Aoki, K., Kim, S.K., Fujii, Y., Yoshida, K., Kataoka, K., Nakagawa, M.M., Inoue, Y., Hirano, T., Shiraishi, Y., Chiba, K., Tanaka, H., Sanada, M., Nishikawa, Y., Amanuma, Y., Ohashi, S., Aoyama, I., Horimatsu, T., Miyamoto, S., Tsunoda, S., Sakai, Y., Narahara, M., Brown, J.B., Sato, Y., Sawada, G., Mimori, K., Minamiguchi, S., Haga, H., Seno, H., Miyano, S., Makishima, H., Muto, M. and Ogawa, S., 2019. Age-related remodelling of oesophageal epithelia by mutated cancer drivers. Nature. 565, 312-317.
Yu, Y.W., Lin, C.H., Chen, S.P., Hong, C.J. and Tsai, S.J., 2000. Intelligence and event-related potentials for young female human volunteer apolipoprotein E epsilon4 and non-epsilon4 carriers. Neurosci. Lett. 294, 179-181.
Zhang, Q., Guo, S., Zhang, X., Tang, S., Shao, W., Han, X., Wang, L. and Du, Y., 2015. Inverse relationship between cancer and Alzheimer's disease: a systemic review meta-analysis. Neurol. Sci. 36, 1987-1994.
Zhang, W.B., Ye, K., Barzilai, N. and Milman, S., 2021. The antagonistic pleiotropy of insulin-like growth factor 1. Aging Cell. 20, e13443.
Zietsch, B.P., Sidari, M.J., Abdellaoui, A., Maier, R., Långström, N., Guo, S., Beecham, G.W., Martin, E.R., Sanders, A.R. and Verweij, K.J.H., 2021. Genomic evidence consistent with antagonistic pleiotropy may help explain the evolutionary maintenance of same-sex sexual behaviour in humans. Nat. Hum. Behav. 5, 1251-1258.
Zou, K., Gong, J.S., Yanagisawa, K. and Michikawa, M., 2002. A novel function of monomeric amyloid beta-protein serving as an antioxidant molecule against metal-induced oxidative damage. J. Neurosci. 22, 4833-4841.

Supplementary discussion

Biological constraint, evolutionary spandrels and antagonistic pleiotropy

David Gems and Carina Kern

Detailed discussion of five examples of antagonistic pleiotropy

Here we consider, for selected examples of antagonistic pleiotropy (AP), the possible constraint that underlies it, and the type of programmatic mechanism involved. For a full list of examples (actual and potential), see Supplementary Table 1.

***AAT1* (α1-antitrypsin)**. This gene, considered briefly in the main discussion, appears to exhibit at least three modes of AP, arising from multiplex constraint.

Elastase degradation of elastin aids migration of neutrophils through tissue to sites of infection, thus aiding in protection against infection but causing tissue injury in the process. This exemplifies a firehose-type costly program, where acute responses to immediate danger contribute to long-term collateral tissue injury. Thus, elastase function is subject to constraint: it is not possible to both optimize tissue migration capacity and minimize collateral tissue injury. This is an organizational constraint of the interconnection type.

Firehose-type detriments involve programmatic injury, including programmatic molecular damage. Injury caused by phagocyte tissue invasion and degranulation is the detrimental component of a costly program. Mechanistically, injury is caused by elastase hyperfunction.

AAT inhibits elastase, which otherwise breaks down elastin. The *AAT1* M allele is the common allele, and MM homozygotes have normal (typical) plasma AAT levels. The Z allele, causes a Glu^{342} to Lys substitution, and ZZ homozygotes show an 84% drop in plasma AAT. In MZ heterozygotes a 17% drop is seen (Listì et al., 2007). ZZ is predicted to increase risk of chronic obstructive disease (COPD) and emphysema, due to increased neutrophil elastase and the resulting increase in tissue injury.

But there is evidence from a Sicilian population that Z allele frequency increases with age, from 3.1% in young controls to 13.3% in centenarians (Listì et al., 2007), suggesting that the Z allele somehow protects against aging. This could reflect protection against cardiovascular disease by the Z allele. The Z allele was found to be less common in young patients affected by acute myocardial infarction (Listì et al., 2007). The ZZ and MZ were also found to be associated with lower blood pressure in coronary artery disease patients, and MZ with reduced risk of ischemic cerebrovascular disease and coronary artery disease (Dahl et al., 2003; Tanash et al., 2020). It has been speculated that neutrophil elastase might break down elastic tissue in the arterial wall, altering the distensibility of the vessel wall in a way that reduces blood pressure and cardiac load (Dahl et al., 2003).

Thus, elevated levels of neutrophil elastase are predicted to increase risk of COPD and emphysema, but may reduce risk of cardiovascular disease and increase lifespan. This is a second potential example of AP exhibited by *AAT1*. Again, elastase function is subject to constraint: it is not possible to both maximize benefits in terms of protective reductions in hypertension, and minimize collateral tissue injury from neutrophil migration in the lung. This is an organizational constraint of the interconnection type. The harm to cardiovascular health arising from wild-type *AAT1* is, according to the Dahl et al. hypothesis, deficiency in a corrective mechanism that reduces hypertension, i.e. hypofunction.

A third possible form of AP relates to the interaction between AAT and elastase. This results in suicide cleavage of AAT and release of a C-terminal 36 amino acid fragment (C-36). There is evidence that C-36 has proinflammatory properties and promotes atherogenesis (Dichtl et al., 2000). Thus, the *AAT1* Z allele, by reducing AAT levels, could reduce atherosclerosis and risk of acute myocardial infarction. In this form of AP, AAT rather than elastase is subject to constraint. It is not possible to both maximize the benefits of inhibiting elastase and the collateral injury that it causes, and minimize the atherogenesis-promoting properties of C-36. This is an organizational constraint of the interconnection type. Mechanistically, injury is caused by AAT (specifically C-36) hyperfunction. The promotion of atherosclerosis by C-36 involves a quasi-program.

***AR* (androgen receptor)**. This gene appears to exhibit at least four modes of AP, arising from multiplex constraint.

AR responds to androgenic hormones, particularly testosterone and dihydrotestosterone. The AR gene has variable numbers of CAG repeats (encoding polyglutamine tracts). AR encoded by alleles with fewer repeats are more responsive to androgens.

In men, shorter CAG repeats increases reproductive fitness in young adults (increased sperm viability, putative enhanced attractiveness to females) (Butovskaya et al., 2015; Dowsing et al., 1999), but in later life promotes benign prostatic hyperplasia (BPH) and prostate cancer (Ingles et al., 1997). If BPH or prostate cancer are androgen-dependent, triggered quasi-programs, then androgen and AR function is subject to asynchronous developmental constraint, since androgens have different effects on the prostate gland in early and late adulthood. In later life, AR is hyperfunctional, and BPH, which increases prostate cancer risk, is the result of a quasi-program.

A second form of AP relates to Kennedy's disease (spinal and bulbar muscular atrophy), a neurodegenerative disease affecting the brainstem and spinal cord. *AR* is X-linked, hence this conditions occurs largely in men. The CAG repeats in *AR* encode polyglutamine tracts which can have a toxic gain-of-function effect, as in Huntington's disease. Shorter CAG tracts reduce risk of Kennedy's disease (La Spada et al., 1991), but increase BPH and risk of prostate cancer (Ingles et al., 1997). Here the constraint involves two different activities of the AR protein: aggregation to promote Kennedy's disease, and hyperfunction to promote BPH. This is an example of *molecular constraint*, i.e. the constraint occurs due to properties of the AR protein itself, rather than of the system the protein affects. Although Kennedy's disease is caused by the AR protein (by its aggregation), this is unrelated to receptor function, so it is not a consequence of hyperfunction. Rather, this is an example of molecular constraint-derived, programmatic molecular damage.

A third form of AP relates to AR action in women. Shorter CAG repeat length reduces breast cancer risk (Rebbeck et al., 1999), but increases ovarian cancer risk (Levine and Boyd, 2001). Androgens inhibit proliferation of mammary epithelia but stimulate that of ovarian epithelia. Here constraint occurs due to differences between tissues in their response to androgens. The promotion of ovarian cancer by short CAG repeat *AR* alleles is likely due to AR hyperfunction, and an androgenic quasi-program in ovarian epithelia (cf AR hyperfunction in the prostate).

A fourth form of AP relates to the likely existence of sexual antagonism in selection for *AR* alleles. For example, shorter CAG length increases male reproductive fitness but also increases ovarian cancer. Here a sexual dimorphism constraint is operative. The programmatic mechanisms involved are as described above.

***Htt* (Huntingtin)**. This gene was initially viewed as an example of a late-acting deleterious mutation without linked benefits, but there is evidence that it exhibits at least two modes of AP.

Huntingtin is a protein of unknown function expressed in many tissues, particularly the brain. The gene contains a series of CAG repeats, encoding a string of glutamine (Q) residues. The number of CAG repeats is variable, in most cases 6-35. Rare alleles with more than 35 repeats (up to ~250 repeats) cause Huntington's disease (HD) because long polyQ tracts promote protein aggregation, which disrupts neuronal function. This severe and fatal neurodegenerative disease has an age of onset typically between 30 and 50 years, and disease-causing alleles are genetically dominant.

The disease-causing *Htt* allele was found to increase fertility by some studies (Shokeir, 1975; Walker et al., 1983) though not all (Kishimoto et al., 1959; Reed and Neel, 1959). How this occurs is unclear, but one possibility is that it is due to behavioral alterations attributable to early stages of neurodegeneration. Disease-causing *Htt* alleles cause increased incidence of hypersexuality (including paraphilias), which has been suggested as an explanation for increased offspring number (Dewhurst et al., 1970), but concrete evidence for this is lacking. There is evidence that the alleles cause bearers to continue to have children at later ages when childbearing in unaffected individuals is more constrained (Shokeir, 1975; Walker et al., 1983), possibly reflecting impairment of judgement. The great American folk singer Woodie Guthrie married 3 times and had 8 children before dying of HD at the age of 55.

As far as cognition and behavior are concerned, drawing a clear line between illness and health is sometimes difficult. Mild (fruste) forms of a variety of neurological and psychiatric disorders can give rise to cognitive and performance enhancement, including bipolar disorder, autism, Tourette syndrome, and even neurosyphilis (Crespi, 2016; Kramer, 1993; Redfield Jamison, 1993; Sacks, 2011). In such cases, a mild functional defect leads to enhanced performance and fitness (here increased fertility), while a more severe functional defect leads to disease.

If disease-causing *Htt* alleles conform to this description, then HD can be understood as the result of an unusual form of hyperfunction in which a program involving a mild and beneficial defect later becomes a pathogenic quasi-program. If correct, this suggests that it is the aggregation-promoting properties of the polyQ tract in Huntingtin rather than an activity arising from Huntingtin function that provides the fitness benefits of disease-causing *Htt* alleles. One possibility is that in its early stages, polyQ aggregation triggers cellular responses (e.g. the UPR) which give rise to some benefits, i.e. a hormetic effect. Another is that mild disturbance of neuronal function, and even subtle neurodegeneration results in functional enhancements. The constraint operative here is of the asynchronous developmental type, leading to a neurodegenerative quasi-program.

In a second form of AP, disease-causing *Htt* alleles increase resistance to several forms of cancer (McNulty et al., 2018; Sorenson et al., 1999). This may be due to increased levels of apoptosis, perhaps due to increased levels of the pro-apoptotic and anti-cancer protein p53 (Eskenazi et al., 2007; Sorenson et al., 1999). According to this view in HD patients increased apoptosis protects against cancer but promotes neuronal apoptosis and neurodegeneration. The possible constraint here relates to p53 and apoptosis, which protect against cancer but cause cell loss. Higher levels of apoptosis provide early life benefit (cancer protection) but long term harm (neurodegeneration). This is an interconnection constraint of the asynchronous developmental type.

There is evidence of purifying (negative) selection at the *Htt* locus (Peng et al., 2007), which is at least consistent with AP at the *Htt* locus (Byars and Voskarides, 2020).

***Xmrk* (*Xiphophorus* melanoma receptor kinase)**. This is a particularly informative case. This gene appears to exhibit at least 4 modes of AP, arising from multiplex constraint.

Xiphophorus is a genus of fish (platyfishes and swordtails), e.g. *X. cortezi*. *Xmrk* is a paralog of the *egfr-b* protooncogene (epidermal growth factor receptor). It contains two activating DNA alterations that make it able to signal without ligand binding, i.e. it is constitutively active, and an oncogene. *Xmrk* is present in some but not all individuals, and is genetically dominant. *Xmrk* causes the spotted caudal (Sc) melanin pattern on the surface of male *X. cortezi*, which is attractive to females (Fernandez and Morris, 2008). It also causes melanoma, which develops due to invasion of melanocytes into the underlying muscle.

Xmrk exhibits AP, promoting fitness by increasing male reproductive success, but shortening lifespan by causing cancer. Because *Xmrk* is an oncogene, fish that lack it have been referred to as wild type (Fernandez and Bowser, 2010). However, this is an example of the disruption paradigm-based fallacy that if a given allele of a gene causes disease, then it is mutant. *Xmrk* promotes reproductive fitness, has existed in the *Xiphophorus* genus for millions of years, and has experienced purifying selection (Fernandez and Bowser, 2010); hence *X. cortezi* individuals with and without *Xmrk* are both wild type.

Also, the melanoma here is of an unusual sort, not involving somatic mutation but rather hyperproliferation of wild-type, oncogene-containing melanocytes: it is wild-type cancer. AP arises due to constraint on both maximizing male reproductive fitness and minimizing risk to male health from melanoma. This is an example of a mixed constraint: that is selective (here sexual selection) and organizational (increased melanocyte proliferation). Male *X. cortezi* with *Xmrk* experience disease due to hyperfunction in the form of a run-on-type quasi-program. Those without *Xmrk* may experience reduced reproductive fitness due to being sexually drab and lacking in decoration, a form of hypofunction.

A second possible form of AP is suggested by possible effects of *Xmrk* on body size. Male *X. cortezi* with *Xmrk* grow larger, which not only further increases attractiveness to females, but also reduces predation (Fernandez and Bowser, 2010). Thus, *Xmrk* increases mortality by causing melanoma as a cost of a sexually selected trait, but also reduces mortality from predation. Here there exists constraint between benefits of sexual selection and natural selection, another form of biological constraint (*S. vs N. selective constraint*). Sexually selected traits often generate costs in terms of natural selection (Zuk and Kolluru, 1998).

The presence of two further forms of AP are suggested by the fact that *Xmrk* has not evolved to fixation in any *Xiphophorus* species or population, implying the presence of balancing selection. One possibility stems from the fact that *Xmrk* can also cause melanoma in females, suggesting possible antagonistic selection: for *Xmrk* in males and against it in females (Fernandez and Morris, 2008). This would be an example of AP arising from sexual dimorphism constraint. Another, theoretical possibility is that *Xmrk* exhibits overdominance, promoting fitness more in heterozygotes, an example of AP arising from allele dosage constraint.

***ADRB2* (beta-2 adrenergic receptor)**. ADRB2 is a receptor for both the hormone epinephrine and the neurotransmitter norepinephrine. ADRB2 is expressed in smooth muscle of the airways and blood vessels and in the CNS. Adrenergic activation of ADRB2 regulates vasomotor tone

and blood pressure, but ADRB also has pulmonary and endocrine functions, and in the CNS. Unsurprisingly, *ADRB2* is highly pleiotropic. In a complex fashion *ADRB2* genotype affects risk of heart disease, hypertension, obesity, COPD, diabetes, asthma, Alzheimer's disease and cancer (Bao et al., 2005; Cagliani et al., 2009; Kulminski et al., 2010). Arg16Gly, Gln27Glu variants improve cognition in the young but reduce it in later life (Bochdanovits et al., 2009), and reduce hypertension in the young but increase it in later life (Bao et al., 2005). They also show opposite effects on longevity in young old and oldest old ages (Kulminski et al., 2010).

ADRB2 has numerous functions, making the mechanisms of AP in this case challenging to decipher. Beta-2 adrenergic signaling promotes memory and learning. Adrenergic activation of ADRB2 regulates vasomotor tone and blood pressure. ADRB2 also stimulates fibroblast and endothelial cell proliferation, and has been linked to inhibition of cellular immunity in cancer (Kulminski et al., 2010). Plausibly, the multiple functions of ADRB2 creates multiplex constraint, such that ADRB2 optimization requires functional compromises that lead to hyperfunction and quasi-programs, or to hypofunction.

Supplemental references

Bao, X., Mills, P.J., Rana, B.K., Dimsdale, D.E., Schork, N.J., Smith, D.W., Rao, F., Milic, M., O'Connor, D.T., Ziegler, M.G., 2005. Interactive effects of common beta2-adrenoceptor haplotypes and age on susceptibility to hypertension and receptor function. Hypertension 46, 301-307.

Bochdanovits, Z., Gosso, F.M., van den Berg, L., Rizzu, P., Polderman, T.J.C., Pardo, L.M., Houlihan, L.M., Luciano, M., Starr, J.M., Harris, S.E., Deary, I.J., de Geus, E.J.C., Boomsma, D.I., Heutink, P., Posthuma, D., 2009. A Functional polymorphism under positive evolutionary selection in ADRB2 is associated with human intelligence with opposite effects in the young and the elderly. Behav. Genet. 39, 15-23.

Butovskaya, M.L., Lazebny, O.E., Vasilyev, V.A., Dronova, D.A., Karelin, D.V., Mabulla, A.Z.P., Shibalev, D.V., Shackelford, T.K., Fink, B., Ryskov, A.P., 2015. Androgen receptor gene polymorphism, aggression, and reproduction in Tanzanian foragers and pastoralists. PLoS One 10, e0136208.

Byars, S.G., Voskarides, K., 2020. Antagonistic pleiotropy in human disease. J. Mol. Evol. 88, 12-25.

Cagliani, R., Fumagalli, M., Pozzoli, U., Riva, S., Comi, G.P., Torri, F., Macciardi, F., Bresolin, N., Sironi, M., 2009. Diverse evolutionary histories for beta-adrenoreceptor genes in humans. Am. J. Hum. Genet. 85, 64-75.

Crespi, B.J., 2016. Autism as a disorder of high intelligence. Front. Neurosci. 10, 300.

Dahl, M., Tybjaerg-Hansen, A., Sillesen, H., Jensen, G., Steffensen, R., Nordestgaard, B.G., 2003. Blood pressure, risk of ischemic cerebrovascular and ischemic heart disease, and longevity in alpha(1)-antitrypsin deficiency: the Copenhagen City Heart Study. Circulation 107, 747-752.

Dewhurst, K., Oliver, J.E., McKnight, A.L., 1970. Socio-psychiatric consequences of Huntington's disease. Br. J. Psychiatry 116, 255-258.

Dichtl, W., Moraga, F., Ares, M.P., Crisby, M., Nilsson, J., Lindgren, S., Janciauskiene, S., 2000. The carboxyl-terminal fragment of alpha1-antitrypsin is present in atherosclerotic plaques and regulates inflammatory transcription factors in primary human monocytes. Mol. Cell. Biol. Res. Commun. 4, 50-61.

Dowsing, A.T., Yong, E.L., Clark, M., McLachlan, R.I., de Kretser, D.M., Trounson, A.O., 1999. Linkage between male infertility and trinucleotide repeat expansion in the androgen-receptor gene. Lancet 354, 640-643.

Eskenazi, B.R., Wilson-Rich, N.S., Starks, P.T., 2007. A Darwinian approach to Huntington's disease: subtle health benefits of a neurological disorder. Med. Hypotheses 69, 1183-1189.

Fernandez, A., Bowser, P., 2010. Selection for a dominant oncogene and large male size as a risk factor for melanoma in the Xiphophorus animal model. Mol Ecol 19, 3114-3123.

Fernandez, A.A., Morris, M.R., 2008. Mate choice for more melanin as a mechanism to maintain a functional oncogene. Proc. Natl. Acad. Sci. U S A. 105, 13503-13507.

Ingles, S.A., Ross, R.K., Yu, M.C., Irvine, R.A., La Pera, G., Haile, R.W., Coetzee, G.A., 1997. Association of prostate cancer risk with genetic polymorphisms in vitamin D receptor and androgen receptor. J. Natl. Cancer Inst. 89, 166-170.

Kishimoto, K., Nakamura, M., Sotokawa, Y., 1959. On population genetics of Huntington's chorea in Japan: a propos de la choree d'Huntington au Japan, Neuropathology. Elsevier, pp. 217-226.

Kramer, P.D., 1993. Listening to Prozac. Penguin.

Kulminski, A.M., Culminskaya, I., Ukraintseva, S.V., Arbeev, K.G., Land, K.C., Yashin, A.I., 2010. Beta2-adrenergic receptor gene polymorphisms as systemic determinants of healthy aging in an evolutionary context. Mech. Ageing Dev. 131, 338-345.

La Spada, A.R., Wilson, E.M., Lubahn, D.B., Harding, A.E., Fischbeck, K.H., 1991. Androgen receptor gene mutations in X-linked spinal and bulbar muscular atrophy. Nature 352, 77-79.

Levine, D.A., Boyd, J., 2001. The androgen receptor and genetic susceptibility to ovarian cancer: results from a case series. Cancer Res. 61, 908-911.

Listì, F., Candore, G., Grimaldi, M.P., Lio, D., Colonna-Romano, G., Orlando, V., Caruso, M., Hoffmann, E., Paolisso, G., Franceschi, C., Caruso, C., 2007. Alpha1-antitrypsin heterozygosity plays a positive role in attainment of longevity. Biogerontology 8, 139-145.

McNulty, P., Pilcher, R., Ramesh, R., Necuiniate, R., Hughes, A., Farewell, D., Holmans, P., Jones, L., Network, REGISTRY Investigators of the European Huntington's Disease Network, 2018. Reduced cancer incidence in Huntington's disease: analysis in the registry study. J. Huntingtons Dis. 7, 209-222.

Peng, J., Redman, C.M., Wu, X., Song, X., Walker, R.H., Westhoff, C.M., Lee, S., 2007. Insights into extensive deletions around the XK locus associated with McLeod phenotype and characterization of two novel cases. Gene 392, 142-150.

Rebbeck, T.P., Kantoff, P.W., Krithivas, K., Neuhausen, S., Blackwood, M.A., Godwin, A.K., Daly, M.B., Narod, S.A., Garber, J.E., Lynch, H.T., Weber, B.L., Brown, M., 1999. Modification of BRCA1-associated breast cancer risk by the polymorphic androgen-receptor CAG repeat. Am. J. Hum. Genet. 64, 1371-1377.

Redfield Jamison, K., 1993. Touched With Fire: Manic-Depressive Illness and the Artistic Temperament. Simon & Schuster.

Reed, T.E., Neel, J.V., 1959. Huntington's chorea in Michigan. 2. Selection and mutation. Am. J. Hum. Genet. 11, 107-136.

Sacks, O., 2011. The Man Who Mistook His Wife for a Hat. Picador.

Shokeir, M., 1975. Investigation on Huntington’s disease in the Canadian Prairies. II. Fecundity and fitness. Clin. Genet. 7, 349-353.

Sorenson, S.A., Fenger, K., Olsen, J., 1999. Significantly lower incidence of cancer among patients with Huntington’s disease. Cancer 6, 1342-1346.

Tanash, H., Ekström, M., Basil, N., Rönmark, E., Lindberg, A., Piitulainen, E., 2020. Decreased risk of ischemic heart disease in individuals with severe alpha 1-antitrypsin deficiency (PiZZ) in comparison with the general population. Int. J. Chron. Obstruct. Pulmon. Dis. 15, 1245-1252.

Walker, D.A., Harper, P.S., Newcombe, R.G., Davies, K., 1983. Huntington's chorea in South Wales: mutation, fertility, and genetic fitness. J. Med. Genet. 20, 12-17.

Zuk, M., Kolluru, G.R., 1998. Exploitation of sexual signals by predators and parasitoids. Q. Rev. Biol. 73, 415–438.
